# Reduced dosage of the chromosome axis factor Red1 selectively disrupts the meiotic recombination checkpoint in *Saccharomyces cerevisiae*

**DOI:** 10.1371/journal.pgen.1006928

**Published:** 2017-07-26

**Authors:** Tovah E. Markowitz, Daniel Suarez, Hannah G. Blitzblau, Neem J. Patel, Andrew L. Markhard, Amy J. MacQueen, Andreas Hochwagen

**Affiliations:** 1 Department of Biology; New York University; New York, NY; United States of America; 2 Whitehead Institute for Biomedical Research; Cambridge, MA; United States of America; 3 Department of Molecular Biology and Biochemistry; Wesleyan University; Middletown, CT; United States of America; National Cancer Institute, UNITED STATES

## Abstract

Meiotic chromosomes assemble characteristic “axial element” structures that are essential for fertility and provide the chromosomal context for meiotic recombination, synapsis and checkpoint signaling. Whether these meiotic processes are equally dependent on axial element integrity has remained unclear. Here, we investigated this question in *S*. *cerevisiae* using the putative condensin allele *ycs4S*. We show that the severe axial element assembly defects of this allele are explained by a linked mutation in the promoter of the major axial element gene *RED1* that reduces Red1 protein levels to 20–25% of wild type. Intriguingly, the Red1 levels of *ycs4S* mutants support meiotic processes linked to axis integrity, including DNA double-strand break formation and deposition of the synapsis protein Zip1, at levels that permit 70% gamete survival. By contrast, the ability to elicit a meiotic checkpoint arrest is completely eliminated. This selective loss of checkpoint function is supported by a *RED1* dosage series and is associated with the loss of most of the cytologically detectable Red1 from the axial element. Our results indicate separable roles for Red1 in building the structural axis of meiotic chromosomes and mounting a sustained recombination checkpoint response.

## Author summary

Meiosis is a specialized cellular division that reduces chromosome copy by half to produce four gametes. To ensure proper chromosome segregation, a meiotic cell must induce DNA double strand breaks, repair them with the homologous chromosome, and fully align the homologs. These aspects of meiosis are dependent on specialized meiotic chromosome axes and the proteins that control this structure, such as Red1 in *S*. *cerevisiae*. Here we analyze the effects of reduced Red1 levels on meiotic processes. Our results suggest that a comparatively small amount of Red1 is sufficient to promote DNA breakage and repair, whereas most of the cytologically detectable Red1 is necessary for controlling the ability of the cell to delay meiotic progression when double strand breaks remain unrepaired.

## Introduction

Meiosis is a specialized developmental process, in which a diploid cell undergoes two chromosomal divisions without an intervening S phase to produce haploid gametes for sexual reproduction. The reduction in ploidy occurs during meiosis I, when homologous chromosomes are segregated. To enable this unique segregation pattern, meiotic cells must identify and physically link homologous chromosome pairs. In most sexually reproducing organisms, such linkages are achieved by meiotic recombination. In addition to increasing genetic variation in the progeny, recombination leads to crossover exchanges that, together with sister chromatid cohesion, stably connect homologous chromosomes [1].

Meiotic crossover formation relies on the controlled introduction and repair of a large number of programmed DNA double-strand breaks (DSBs). DSBs are formed in a non-random manner across the genome by the conserved topoisomerase-like enzyme Spo11 [2–7]. In the budding yeast *Saccharomyces cerevisiae*, 160 DSBs are estimated to occur on average per meiosis [8]. Following DSB formation, nucleolytic processing releases Spo11 from break ends along with short covalently-linked oligonucleotides [9]. Exonucleases resect the break ends to produce 3’ single-stranded DNA (ssDNA) tails [10] that are used by the recombinases Rad51 and Dmc1 to preferentially invade the homologous chromosomes [11,12]. Some of the resulting strand-invasion intermediates are stabilized and processed to produce crossovers [13–17].

Meiotic recombination occurs in the context of highly conserved chromosome architecture, characterized by linear arrays of chromatin loops anchored to a proteinaceous axis known as the axial element [18–20]. As meiotic prophase progresses, axial elements of homologous chromosomes in many organisms co-align with the help of transverse filament proteins, such as Zip1 in yeast and Sycp1 in mice [21]. The axial elements subsequently form the lateral elements of the synaptonemal complex (SC), a ladder-like structure connecting homologous chromosome pairs in the later stages of meiosis [21]. Like the rest of the SC, axial elements are defined cytologically; they appear as electron dense linear structures by electron microscopy and can be visualized by immunofluorescence analysis of proteins localizing to these structures [20,22–26]. The structural axes of meiotic chromosomes are also defined functionally, with mutants that lose axial integrity exhibiting numerous defects in meiotic recombination, including reduced DSB formation, aberrant DSB repair, and a loss of DSB surveillance [18,27,28]. These defects lead to inviable gametes in a variety of model organisms [20] and are associated with male infertility and premature ovarian failure in human patients [29,30].

The molecular structure of the axial elements is only beginning to be understood. In *S*. *cerevisiae*, the axial element comprises a specialized meiotic cohesin complex, containing the meiosis-specific subunit Rec8 [23], as well as the meiotic proteins Red1 and Hop1 [22,24]. Rec8-cohesin is thought to form the chromatin anchor that recruits Red1 and Hop1 to axis-attachment sites along chromosomes [31,32]. Moreover, electron microscopy studies indicate that only Rec8-cohesin is essential for axial element formation, as *red1* and *hop1* mutants still exhibit dark-staining linear structures [23,33,34]. These structures, however, appear fragmented and irregular, suggesting that *RED1* and *HOP1* contribute to axial element formation or stability.

*RED1* and *HOP1* exert essential regulatory functions in the context of the axial element. Deletion of *RED1* or *HOP1* reduces DSB levels to 10–30% of wild type [12,35–39], likely because of the role of these genes in recruiting essential DSB factors near DSB hotspots [31]. Moreover, the DSBs that do form in *red1* and *hop1* mutants are associated with unusually long resection tracts [40,41]. Loss of *RED1* or *HOP1* function also causes severe synapsis defects [33,34], although linear stretches of the central SC component Zip1 remain detectable in some *red1* mutant cells [24]. Finally, *red1* and *hop1* mutants fail to activate the meiotic checkpoint network [42,43], which helps block repair from the sister chromatid to bias repair events toward the homologous chromosome [44,45], and acts to arrest cells in meiotic prophase in response to unrepaired DSBs [27,38,46,47].

Surprisingly, mutants lacking *REC8* recapitulate only some of the phenotypes of *red1* and *hop1* mutants. Although *rec8Δ* mutants are unable to synapse [23] and exhibit excessive resection [41], their defects in DSB formation are more nuanced than in *red1* and *hop1* mutants, affecting some chromosomal regions while sparing others [32,48,49]. In addition, *rec8Δ* mutants are proficient in mounting a meiotic checkpoint response [23,50]. This disparity may be explained by the fact that Red1 and Hop1 are recruited to a limited number of chromosomal regions in the absence of Rec8 [31,32]. Although their distribution pattern is highly abnormal, Hop1- and Red1-rich regions exhibit close to wild-type DSB levels [32,48,49], implying that the axis-associated DSB and checkpoint activities remain functional in these regions.

Red1 and Hop1 recruitment to the axial element is affected in mutants of the condensin complex. The condensin complex is a conserved regulator of chromosome architecture that localizes to axial elements and functions in chromosome compaction and removing cohesin from chromosomes at the end of meiotic prophase [51,52]. The intensity of Red1 and Hop1 on chromosome spreads is decreased in temperature-sensitive condensin mutants [51], and nearly undetectable in the meiosis-specific condensin allele *ycs4S*, which is linked to a C-terminal 12xMYC tag on the condensin subunit Ycs4 [51]. The reason for the more severe meiotic defects of *ycs4S* has remained unclear, in particular as other condensin functions, including cell survival and chromosome compaction, appear unaffected by this allele [51].

Here, we show that the *ycs4S* allele is in linkage with a promoter mutation in the nearby *RED1* gene that reduces *RED1* expression to about 25% of wild-type levels and explains most, if not all, *ycs4S* phenotypes. Intriguingly, the reduced Red1 levels cause a differential loss of *RED1* activities. Analysis of a Red1 dosage series including the *ycs4S* allele shows that DSB formation, deposition of the synapsis protein Zip1, and spore viability are all buffered against substantial reductions in Red1 levels. By contrast, the ability to maintain a checkpoint arrest in meiotic prophase is very sensitive to Red1 dosage and abolished in the *ycs4S* mutant. These data suggest separable activities of Red1 in regulating axis integrity and the maintenance of checkpoint activity, and imply that the majority of Red1 is primarily involved in the maintenance of meiotic checkpoint activity.

## Results

### *RED1* expression is reduced in *ycs4S* mutants

**Fig 1:**
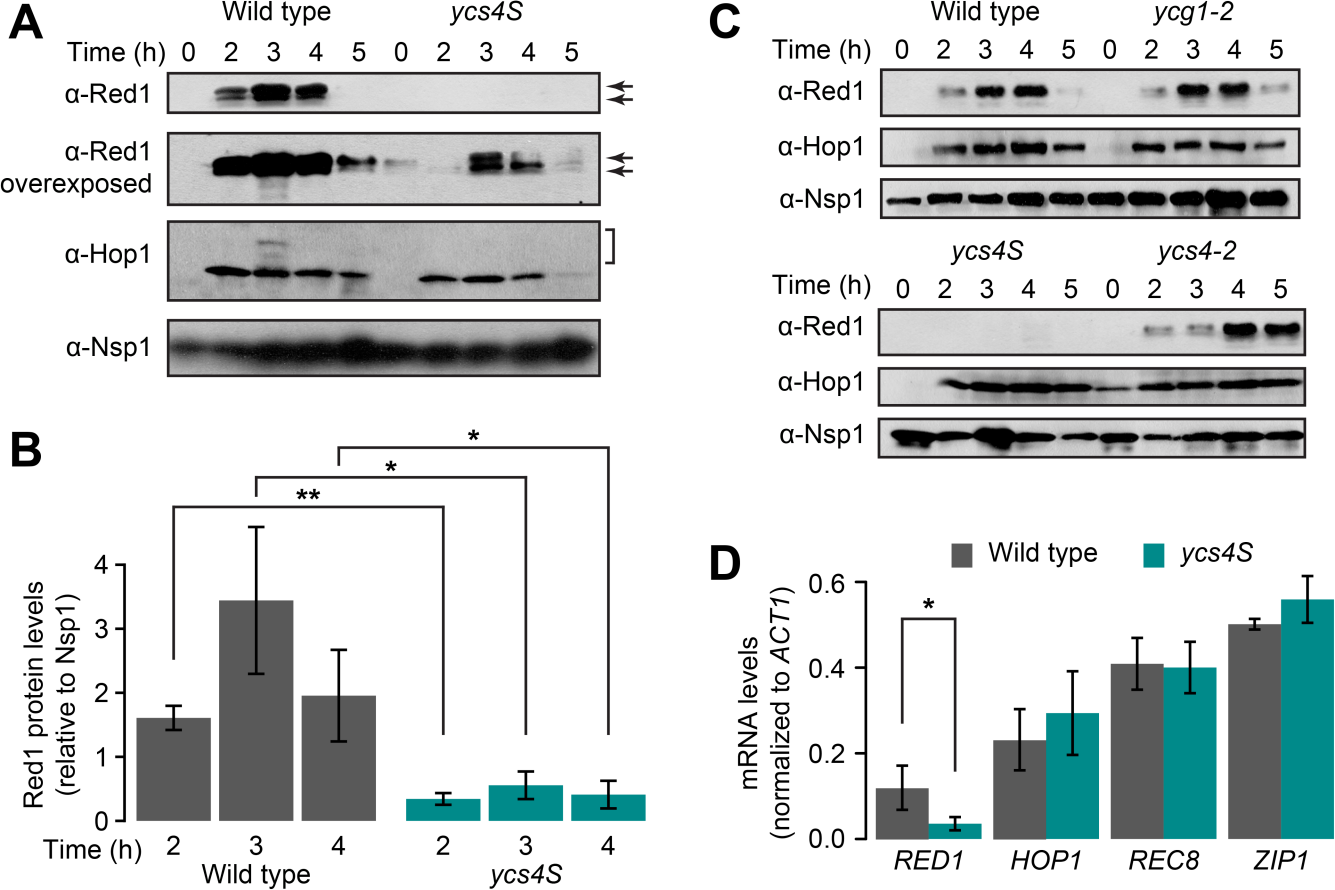
*ycs4S* mutants have decreased levels of Red1. **(A)** Red1 and Hop1 protein levels of whole-cell extracts from wild type (H7797) and *ycs4S* (H7011) as determined by western blotting at the indicated time points after meiotic induction. Arrows indicate the two Red1 bands. Bracket indicates positions of phosphorylated Hop1. Nsp1 was used as loading control. **(B)** Fluorescence-based quantitative measurement of total Red1 protein levels in wild type and *ycs4S* relative to Nsp1 at 2h, 3h, and 4h, n = 3. Error bars: S.E.M. *: p-value < 0.05, **: p-value < 0.005, paired Student *t*-test. **(C)** Western analysis of Red1 and Hop1 protein levels in wild-type, *ycg1-2* (H8632), *ycs4S*, and *ycs4-2* (H8601) strains. Nsp1 was used as loading control. *ycg1-2* and *ycs4-2* cultures were shifted to 34°C 1 hour after meiotic induction. **(D)** *RED1*, *HOP1*, *REC8*, and *ZIP1* mRNA levels at 2h were measured by RT-qPCR from both wild-type (grey) and *ycs4S* (cyan) extracts, and normalized against *ACT1*, n = 3. Error bars: S.D. *: p-value < 0.05, Student *t*-test.

The low Red1 and Hop1 signals localizing to meiotic chromosomes of *ycs4S* mutants [51] prompted us to test whether total axis protein levels are reduced by this allele. To this end, wild-type and *ycs4S* cells were harvested in a synchronous meiotic time course, and the levels of Red1 and Hop1 were analyzed by western blotting. We found that total Hop1 levels are similar in both strains, although we noted a reduction in the slower migrating, phosphorylated forms of Hop1 [53] in the *ycs4S* mutant (Fig 1A). By contrast, Red1 protein levels are strongly reduced in the *ycs4S* mutant relative to wild-type meiotic cells. Quantitative western analyses showed that *ycs4S* mutants express on average approximately 20% of the Red1 protein levels of wild type during meiotic prophase (Fig 1B). This decrease in Red1 protein levels differentiates the *ycs4S* mutant from the temperature-sensitive *ycg1-2* and *ycs4-2* condensin mutants, which do not exhibit a discernable reduction of Red1 levels at the restrictive temperature of 34°C (Fig 1C).

Coincident with the decrease in Red1 protein in the *ycs4S* mutant is a reduction of the slower migrating band, which is the result of Red1 phosphorylation [54] (compare late time points in Fig 1A). The biased loss may be the result of preferential degradation of phosphorylated Red1 in the *ycs4S* mutant or reflect reduced phosphorylation of Red1 as a result of reduced Red1 levels.

To test if the loss of Red1 abundance occurs at the transcriptional or post-transcriptional level, we analyzed relative *RED1* mRNA levels at the 3-hour time point by qRT-PCR. These measurements revealed that *RED1* mRNA levels in *ycs4S* mutants are about 25% of wild-type levels (p < 0.05; Student *t*-test, Fig 1D). By contrast, no significant difference was observed for the mRNA levels of the axial element components *HOP1* and *REC8*, or the transverse filament component *ZIP1* (p = 0.79, p = 0.43, and p = 0.10, respectively). Together, these data indicate that the *ycs4S* allele is associated with a specific loss of *RED1* mRNA expression.

### Increasing Red1 levels rescues the spore inviability of *ycs4S* mutants

**Fig 2:**
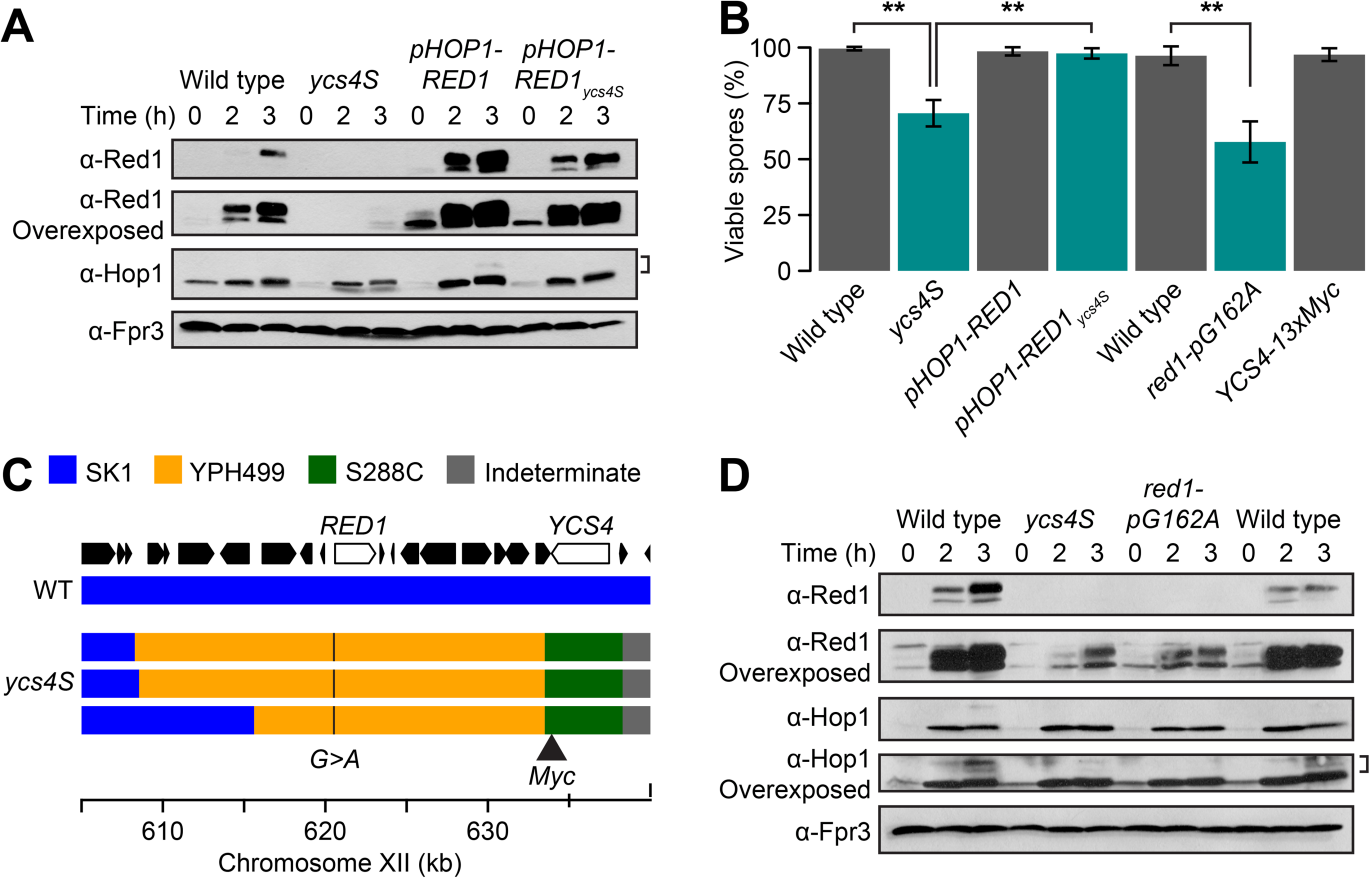
*ycs4S* mutants have a mutation in the promoter of *RED1*. **(A)** Red1 and Hop1 protein levels in wild type (H7797), *ycs4S* (H7011), *pHOP1-RED1* (H8849), and *pHOP1-RED1*_*ycs4S*_ (H8850) strains. Phosphorylated Hop1 indicated by bracket. Fpr3 was used as loading control. **(B)** Spore viability of wild-type, *ycs4S*, *pHOP1-RED1*, *pHOP1-RED1*_*ycs4S*_, and *YCS4-13xMYC* (H9077) strains, as well as *red1-pG162A* (H9048) mutant strain and a matched control carrying only a marker insertion at position +400 upstream of the *RED1* ORF (H9049), n>100. Error bars: S.D. **: p-value: < 0.005, Student *t*-test. (**C**) Schematic of the introgressed region in the *ycs4S* mutant. The genetic background of three separate lines was determined across a 35kb region of chromosome XII, which includes the *RED1* and *YCS4* genes. This was compared to a wild type SK1 background. Regions were color-coded as SK1 (blue), YPH499 (yellow), S288C (green), and indeterminate (grey). Positions of the *12xMYC* tag and the *G162A* SNP in the promoter of *RED1* are indicated. **(D)** Red1 and Hop1 protein levels in wild type and a *ycs4S* mutant, as well as a *red1-pG162A* mutant strain and its matched control. Fpr3 was used as loading control.

Because *ycs4S* differs from other condensin mutants, both in its ability to compact its chromosomes [51] and in its decreased concentrations of Red1 protein, we hypothesized that the meiotic phenotypes displayed by *ycs4S* mutants could be primarily caused by low Red1 expression levels as opposed to a direct effect of condensin malfunction. To test this possibility, we placed *RED1* under the control of the *HOP1* promoter, which is unaffected in the *ycs4S* mutant (Fig 1D). Western blot analysis indicated that the *pHOP1-RED1* construct leads to an overexpression of Red1, although this effect was less obvious in the *ycs4S* mutant (Fig 2A). In addition, Hop1 levels were slightly higher, including an increase in Hop1 phosphorylation signal (Fig 2A, bracket). Importantly, the *pHOP1-RED1* construct completely rescued the spore viability defect of *ycs4S* cells (Student *t*-test: p-value = 1.1 x 10^−4^, Fig 2B).

The ability of the *pHOP1-RED1* construct to rescue the *ycs4S* spore viability defect indicates that the *RED1* promoter has a role in causing the *ycs4S* phenotypes. This initially puzzling result is explained by the fact that the *YCS4* gene (*YLR272C*) is located in close proximity to *RED1* (*YLR263W*), with the *RED1* promoter being separated from the C-terminal *12xMYC* tag of *ycs4S* by less than 14kb. Sequence analysis revealed approximately 100 SNPs across this region that differ from the SK1 strain background and reflect the fact that the *YCS4-12xMYC* construct was introgressed from YPH499 into SK1 (Fig 2C) [51]. The preservation of YPH499-derived SNPs throughout this region over multiple crosses indicates a close genetic linkage between *RED1* and *YCS4*.

We identified a single SNP in the *RED1* promoter of the *ycs4S* mutant, a change from G to A at position -162 from the *RED1* open reading frame. This SNP overlaps a conserved residue of a *URS1* consensus site for the transcriptional regulator Ume6 [55], which is required for wild-type levels of *RED1* expression [56]. Disruption of the corresponding residue along with a second residue in the *URS1* sequence of the meiotic *SPO13* gene causes a 6-fold reduction in expression [57]. Introduction of the YPH499 SNP into an otherwise wild-type SK1 strain resulted in decreased Red1 protein levels (Fig 2D, S1A Fig) and spore viability defects (Student *t*-test: p-value = 1 x 10^−3^, Fig 2B) comparable to those of *ycs4S* mutants. Introduction of a *13xMYC* tag at the C-terminus of *YCS4* had no effect on spore viability (Student *t*-test: p-value = 0.104, Fig 2B). These findings together with the rescue mediated by the *HOP1* promoter suggest that the *ycs4S* phenotypes are caused by a linked mutation in the *RED1* promoter that causes low *RED1* expression. We note that providing the YPH499 genome *in trans* in a SK1/YPH499 hybrid diploid did not improve the spore viability defects caused by the *red1-pG162A* mutation (S1B Fig), indicating that YPH499 has not adapted to this mutation. For the remainder of this paper, we will refer to the introgressed YPH499 genomic fragment containing the *YCS4-12xMYC* construct and the *red1-pG162A* mutation as *red1*_*ycs4S*_.

### Red1 and Hop1 are recruited to axis attachment sites in *red1*_*ycs4S*_ mutants

**Fig 3:**
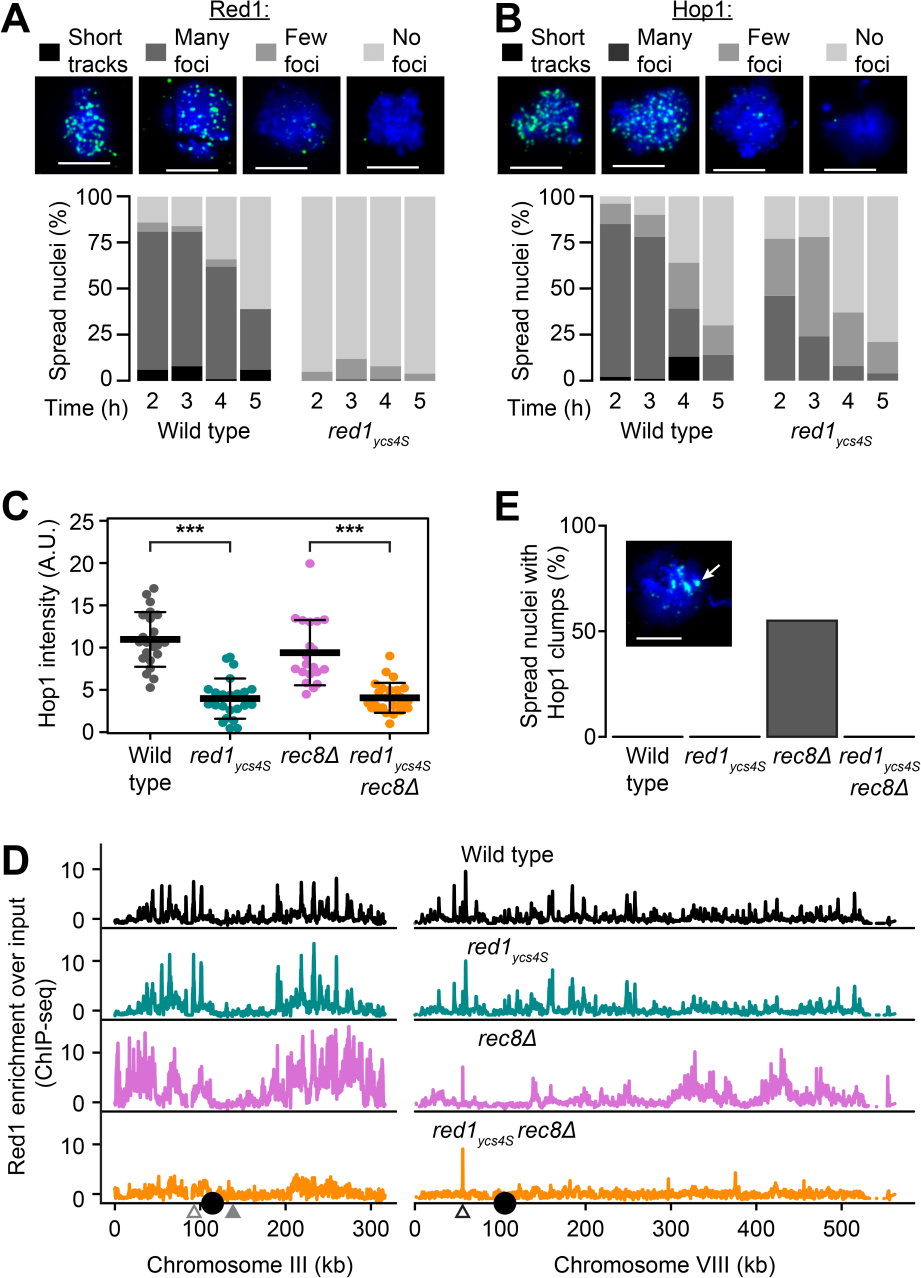
Decreased deposition but normal distribution of axis proteins in *red1*_*ycs4S*_ mutants. **(A)** Red1 binding and **(B)** Hop1 binding was analyzed on chromosome spreads from wild-type (H7797) and *red1*_*ycs4S*_ (H7011) strains, n = 100 per time point. Patterns were classified into four categories: “No foci”, “Few foci” (less than 20 detectable by eye over background), “Many foci”, or “Short Tracks” (indicative of chromosome axes). Scale bars are 5μm. **(C)** Hop1 intensity values of nuclei spread at 2h after meiotic induction. Focus intensity across an entire spread was normalized against background intensity of an area of identical size. Average intensity is indicated for wild-type (grey), *red1*_*ycs4S*_ (cyan), *rec8Δ* (pink, H5187), and *red1*_*ycs4S*_ *rec8Δ* (orange, H7661) strains, n = 20. Error bars: S.D. ***: p-value < 10^−5^, Wilcox test. **(D)** Red1 chromosomal localization at 3h determined by ChIP-seq in wild-type (black, H119), *red1*_*ycs4S*_ (H7011), *rec8Δ* (H7660 & H7772), and *red1*_*ycs4S*_ *rec8Δ* (H7661) strains on chromosomes III and VIII, n = 2. Large black circles indicate the positions of the centromeres. Triangles indicate positions analyzed by ChIP-qPCR in S3B Fig. **(E)** Percentage of spreads at 2h from time course in **(C)** exhibiting clumps of Hop1 (arrow in example image), n = 100.

If the low levels of Red1 protein in the *red1*_*ycs4S*_ mutant are responsible for its axis assembly defect, chromosomal levels of Red1 and Hop1 should be diminished but still distributed at the expected sites along meiotic chromosomes. To test this prediction, we probed Red1 and Hop1 recruitment in *red1*_*ycs4S*_ mutants using immunofluorescence and ChIP-seq analysis. Consistent with published results [51], we found that Red1 and Hop1 levels on chromosome spreads are greatly reduced and no cytological axis structures are observed in *red1*_*ycs4S*_ mutants (Fig 3A and 3B). Notably, whereas Red1 signals are nearly undetectable, nuclei with discernible Hop1 foci still exist. However, the intensity of total Hop1 signal on chromosomes in these nuclei was two-fold lower than in wild-type nuclei (Wilcoxon Sign Rank Test: p-value = 3.9 x 10^−10^, Fig 3C). The incongruity in the amount of Hop1 versus Red1 foci detected in our studies may be the result of different antibody sensitivities. Alternatively, given that Red1 is required for Hop1 recruitment to chromosomes [24,32], it may indicate that multiple Hop1 proteins are recruited per Red1 protein. Importantly, although no axis structures were observed cytologically, ChIP-seq analysis indicated that Red1 and Hop1 are nevertheless recruited in a wild-type pattern to meiotic chromosomes (Fig 3D, S2A Fig, Spearman correlation: 0.87 and 0.66, respectively). We note that the apparently similar signal intensities of wild-type and *red1*_*ycs4S*_ profiles are a consequence of the necessary internal normalization, which precludes comparison of absolute intensities between ChIP-seq datasets [58]. Accordingly, ChIP-qPCR analysis of several loci revealed a general decrease in Red1 binding in the *red1*_*ycs4S*_ mutants (S3B Fig). Together, these analyses show that axis protein binding is reduced in *red1*_*ycs4S*_ mutants, but that the low chromosomal amounts of Red1 and Hop1 are distributed normally to axis recruitment sites, suggesting that their chromosomal binding *per se* is unaffected.

To determine if reducing the Red1 levels in *red1*_*ycs4S*_ mutants affects cohesin-independent recruitment of axis proteins, we introduced a *rec8Δ* mutation. ChIP-seq analysis revealed that the patterning of Red1 and Hop1 is highly concordant in *red1*_*ycs4S*_ *rec8Δ* mutants, a characteristic also observed in *rec8Δ* mutants [32] (Fig 3D, S2A Fig, Spearman correlation: 0.94 and 0.93, respectively). However, in the *red1*_*ycs4S*_ *rec8Δ* mutant, the genomic regions with relative enrichment of Hop1 and Red1 exhibited a substantial dampening relative to the other strains, even though Hop1 and Red1 protein levels are not substantially different from the *red1*_*ycs4S*_ strain (S4A and S4B Fig). Quantification of total chromosome-associated Hop1 signal on chromosome spreads indicated no further loss of axis proteins in the *red1*_*ycs4S*_ *rec8Δ* double mutant compared to the *red1*_*ycs4S*_ mutant (Wilcoxon Sign Rank Test: p-value = 0.66, Fig 3C), but this result is likely caused by the already low signal in the *red1*_*ycs4S*_ mutant. Indeed, ChIP-qPCR analysis revealed a further loss of Red1 enrichment in the double mutant (S3A and S3B Fig). Some binding of Hop1 and Red1 likely remains even in the *red1*_*ycs4S*_ *rec8Δ* mutant, especially on chromosome III, as indicated by a general enrichment compared to a mock ChIP-seq experiment (S2B and S2C Fig) and supported by ChIP-qPCR analysis (S3B Fig). In addition, a few sites with strong Hop1 and Red1 enrichment persist in the double mutant (e.g. Chr VIII, open triangle, Fig 3D, S2A, S2B, S3A and S3B Figs), but their significance is unclear as they are not associated with any obvious chromosomal landmarks.

Analysis of Hop1 signal on chromosome spreads also indicated that the *red1*_*ycs4S*_ allele rescues a characteristic axis defect of *rec8Δ* mutants. While *rec8Δ* mutants form distinctive clumps of Hop1 and Red1 on chromosomes spreads [23], *red1*_*ycs4S*_ *rec8Δ* mutants do not (Fig 3E, S4C Fig). These data indicate that these clumps are dependent on Red1 protein abundance.

### No checkpoint arrest in *red1*_*ycs4S*_ mutants

**Fig 4:**
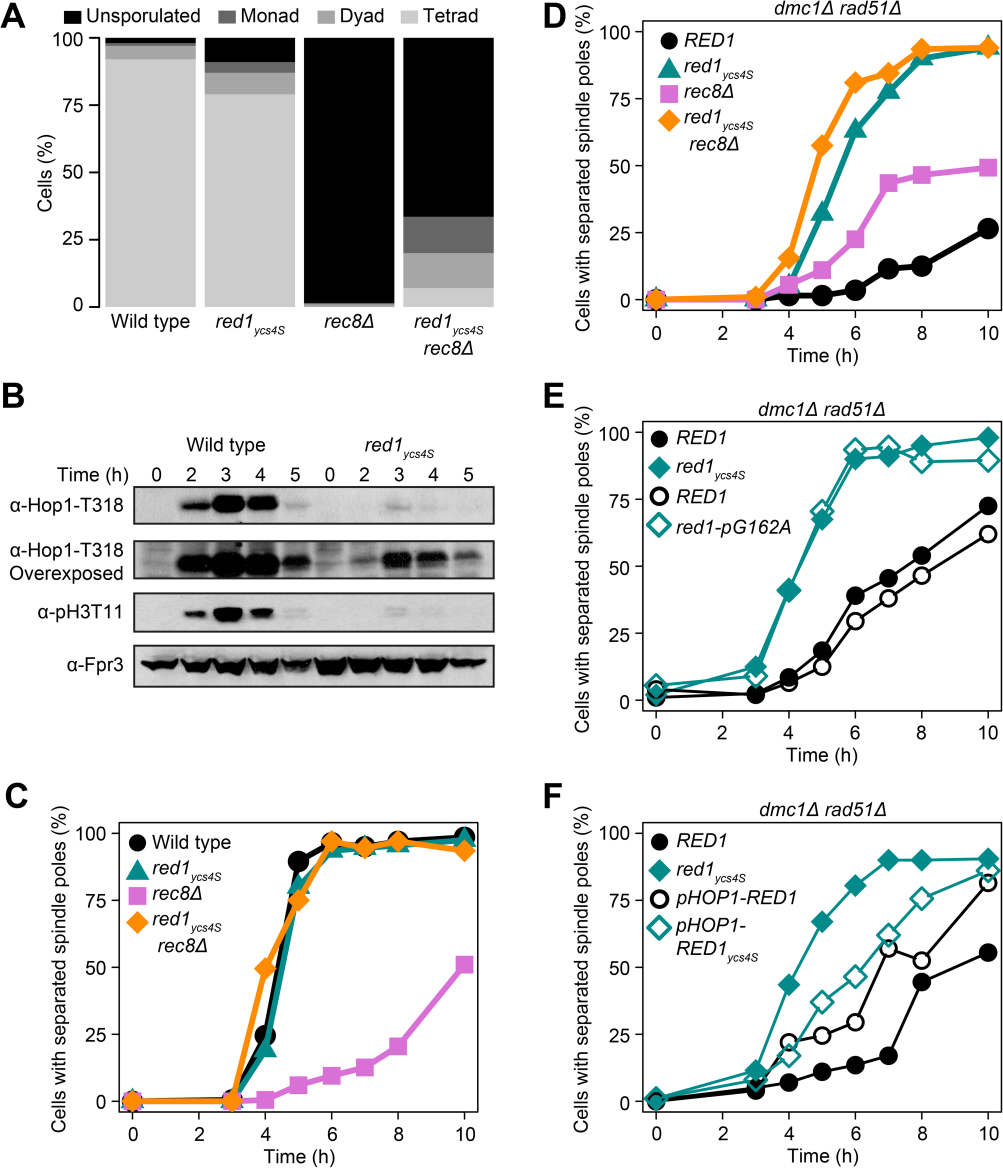
*red1*_*ycs4S*_ mutants lack a sustained meiotic DNA damage checkpoint arrest. **(A)** Sporulation efficiency of *red1*_*ycs4S*_ (H7011), *rec8Δ* (H5187), and *red1*_*ycs4S*_ *rec8Δ* (H7661) mutants relative to wild type (H7797), n = 200. **(B)** Western analysis of phosphorylation levels of Hop1-T318 and H3-T11 from wild-type and *red1*_*ycs4S*_ cells. Fpr3 was used as loading control. **(C)** Spindle pole separation in fixed cells measured by anti-tubulin immunofluorescence of wild type (black circle), *red1*_*ycs4S*_ (cyan triangle), *rec8Δ* (pink square), and *red1*_*ycs4S*_ *rec8Δ* (orange diamond), n = 200. Spindle pole separation is an indication of progression out of meiotic prophase. **(D)** Spindle pole separation in *red1*_*ycs4S*_ (H7088), *rec8Δ* (H7161), and *red1*_*ycs4S*_ *rec8Δ* (H6589) mutants and their control (H7076) in a repair-deficient *dmc1Δ rad51Δ* background, n = 200. **(E)** Spindle pole separation of wild type (black closed circle), *red1*_*ycs4S*_ (cyan closed triangle), *red1-pG162A* (H9080, cyan open triangle) and its matched control (H9078, black open circle) strains in a *dmc1Δ rad51Δ* background to measure checkpoint activity, n = 200. **(F)** Spindle pole separation of wild type (black closed circle), *red1*_*ycs4S*_ (cyan closed triangle), *pHOP1-RED1* (H8851, black open circle), and *pHOP1-RED1*_*ycs4S*_ (H8852, cyan open triangle) strains in a *dmc1Δ rad51Δ* background to measure checkpoint activity, n = 200.

During our analysis of the *red1*_*ycs4S*_ *rec8Δ* mutant, we noticed that these cultures produce a substantial number of spores (Fig 4A). This behavior is in contrast to *rec8Δ* single mutants, which arrest in meiotic prophase and fail to sporulate due to defects in DSB repair [23,50], suggesting that *red1*_*ycs4S*_ *rec8Δ* mutants are unable to mount a sustained checkpoint-mediated arrest response. Consistent with this notion, phosphorylation of Hop1, one of the earliest markers of recombination checkpoint activation [45], and phosphorylation of histone H3 threonine 11 (H3T11), a marker for activity of the downstream checkpoint kinase Mek1 [45,59,60], are reduced in *red1*_*ycs4S*_ cells (Fig 4B).

To investigate the kinetics of this arrest bypass, we followed the rate of meiotic spindle pole separation, which initiates after cells exit meiotic prophase [61]. In a *rec8Δ* mutant, few cells produced separated spindle poles as seen previously [49], whereas *red1*_*ycs4S*_ mutants formed spindles at the same rate as wild type (Fig 4C). Wild-type kinetics of spindle pole separation were also observed in *red1*_*ycs4S*_ *rec8Δ* double mutants, indicating the reduced levels of Red1 in the *red1*_*ycs4S*_ mutant allow a complete bypass of the *rec8Δ* checkpoint arrest (Fig 4C).

We considered two non-exclusive explanations for this bypass. First, *red1*_*ycs4S*_ *rec8Δ* may be able to repair DSBs using an alternative pathway. This possibility is supported by the fact that the barrier to sister chromatid repair is weakened in *red1*_*ycs4S*_ mutants [51,62]. Second, the signaling response triggering the arrest could be weakened by the *red1*_*ycs4S*_ allele, either by reduced DSB initiation or poor signal transduction.

To investigate arrest signaling without the confounding effects of DSB repair, we analyzed spindle pole separation in a *dmc1Δ rad51Δ* background, which prevents essentially all homologous meiotic DNA repair [63]. We confirmed that *red1*_*ycs4S*_ leads to a similar reduction in Red1 levels in the presence of these mutations (S5A and S5B Fig). Importantly, consistent with the model that the reduced Red1 levels interfere with checkpoint signaling, the *dmc1Δ rad51Δ red1*_*ycs4S*_ mutant completely bypassed the checkpoint arrest and proceeded through meiosis at a rate similar to wild-type cells (Fig 4D). This defect is specifically caused by the *red1-pG162A* mutation, as introduction of the SNP is sufficient to cause a bypass (Fig 4E). Moreover, the bypass is partially rescued by increasing *RED1* expression (*pHOP1-RED1*_*ycs4S*_; Fig 4F), indicating that reduced *RED1* levels are responsible for the checkpoint defects. We note that the *pHOP1-RED1* construct itself caused a slight defect in arrest activity, possibly due to *RED1* overexpression, which is known to affect exit from meiotic prophase [64]. Furthermore, because *dmc1Δ rad51Δ* mutants do not completely arrest like *dmc1Δ* mutants [63], we also confirmed that the *red1-pG162A* mutation was able to bypass the arrest of a *dmc1Δ* mutant (S5C Fig). These data indicate that arrest signaling is defective in *red1*_*ycs4S*_ mutants.

### *red1*_*ycs4S*_ mutants produce sufficient DSBs and resection to arrest

**Fig 5:**
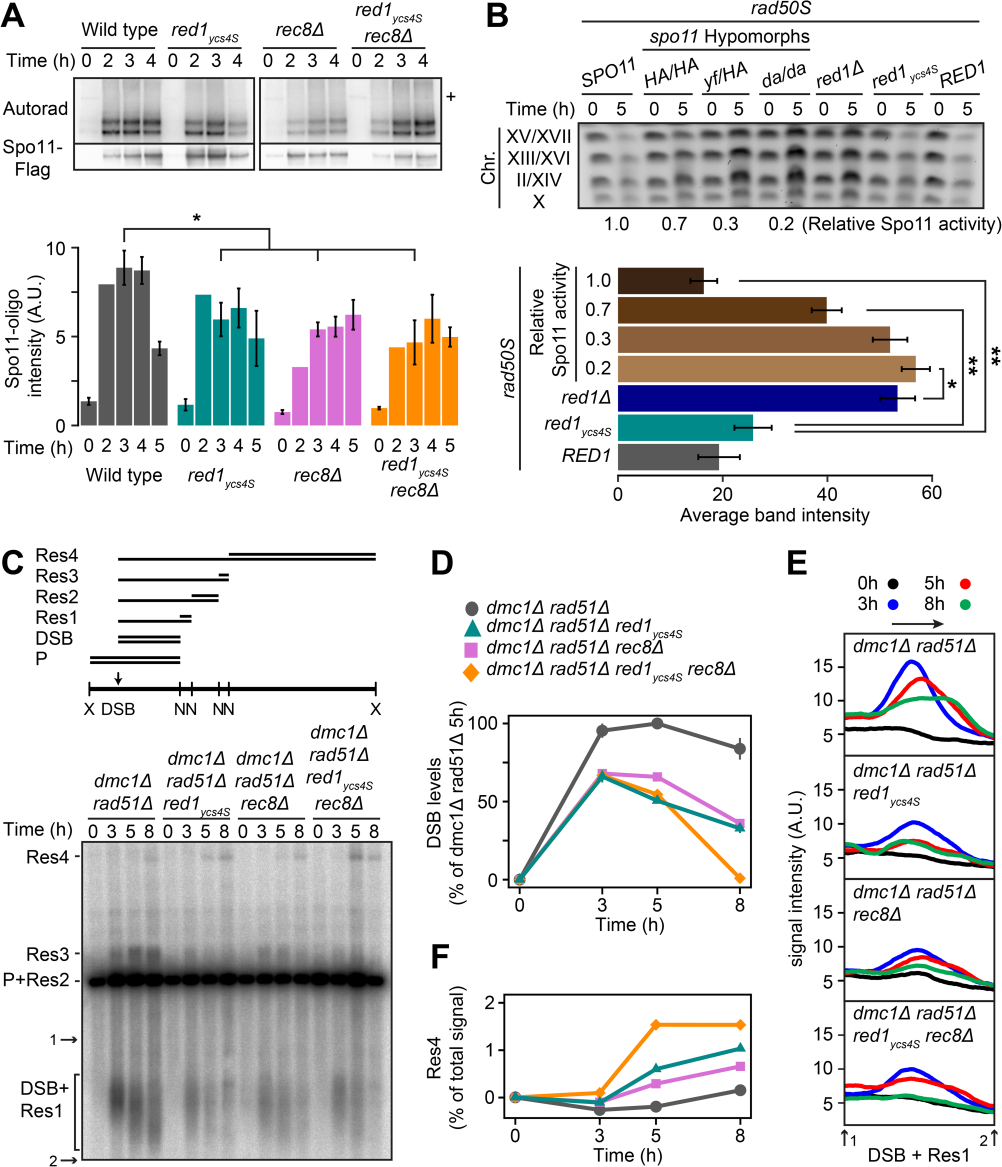
Lack of checkpoint arrest in *red1*_*ycs4S*_ is not caused by insufficient DSBs or a lack of sustained resection. **(A)** Measurement of Spo11-oligonucleotide complexes as a measure of DSB level independent of resection or repair. Experiments were completed in wild-type (H9082), *red1*_*ycs4S*_ (H9084), *rec8Δ* (H9083), and *red1*_*ycs4S*_ *rec8Δ* (H9085) strains. Upper panel: Representative autoradiograph of Spo11-oligo complex and measure of total Spo11 protein by anti-Flag western. Plus sign indicates non-specific band. Lower panel: Quantification of Spo11-oligo complex bands (background normalized), n = 4 for 0, 3, and 4h, n = 3 for 5h. Error bars: S.E.M. *: p-value < 0.05, paired Student *t*-test. **(B)** Upper panel: Ethidium bromide staining showing the levels of seven intact chromosomes in a *red1Δ* (H8058), *red1*_*ycs4S*_ (H8096), and control (H8099) in a *rad50S* background compared to strains with defined DSB levels: 100% (H9202), 70% (H9203), 30% (H9204), and 20% (H9205) [65,66], see S1 Table. Lower panel: Levels of chromosome breakage quantified from top three bands above, n = 2. Error bars: S.E.M. *: p-value < 0.05, **: p-value < 0.005, paired Student *t*-test. **(C)** Upper panel: Schematic of the *CCT6* hotspot indicating uncut parental band (P), DSB fragment length (DSB), and resection products (Res1-4) when genomic DNA is cut with *Xho*I (X) and *Nsi*I (N). Lower panel: DSB and resection products at the *CCT6* hotspot formed after meiotic synchronization in *red1*_*ycs4S*_ (H7088), *rec8Δ* (H7161), and *red1*_*ycs4S*_ *rec8Δ* (H6589) mutants and their control (H7076) in a *dmc1Δ rad51Δ* background. Arrows 1 and 2 were used as anchors to measure signal accumulation in **(E)**. **(D)** DSB levels as a percent of *dmc1Δ rad51Δ* at the *CCT6* hotspot, n = 2 for *dmc1Δ rad51Δ* and *dmc1Δ rad51Δ red1*_*ycs4S*_. Error bars: range. **(E)** Linescans of DSB band from gel in **(C)**. Arrow indicates accumulation of faster migrating bands over time in *dmc1Δ rad51Δ*. **(F)** Rate of accumulation of signal at Res4 as a percent of lane total. Values have been normalized against signal of all bands in the lane and followed by subtraction of the value at 0h.

Our analysis showed that deletion of *REC8* also allowed a partial bypass of the arrest expected in the *dmc1Δ rad51Δ* mutants (Fig 4D), which has been attributed to a reduced number of DSBs [50]. To investigate if reduced DSBs might explain the bypass of the *dmc1Δ rad51Δ* arrest, we determined total DSB activity in *red1*_*ycs4S*_ mutants by immunoprecipitating Spo11 and quantifying the amount of associated oligonucleotides as a measure of Spo11 activity [9,65]. This analysis revealed that maximal DSB levels in *red1*_*ycs4S*_ mutants are reduced by approximately 20% compared to wild type (Fig 5A), in line with Southern measurements at individual hotspots [51,62]. To further support this measurement, we compared chromosome breakage in *red1*_*ycs4S*_ mutants to a collection of three *spo11* mutants with defined DSB activities [65,66]. Quantification of several full-length chromosomes after pulsed-field gel electrophoresis (PFGE) revealed that *red1*_*ycs4S*_ mutants undergo meiotic chromosome breakage at levels significantly below the 100% of wild type but significantly above the ~70% of DSB activity of the *spo11-HA* allele (paired Student *t*-test: p-value = 3 x 10^−4^ and 6.8 x 10^−5^ respectively, Fig 5B). *red1Δ* mutants exhibited ~30% of wild-type breakage levels. These trends were also observed when measuring DSB levels on chromosome XVI by Southern blotting (S6A and S6B Fig).

Importantly, Spo11-oligo analysis indicated that total DSB levels in the *red1*_*ycs4S*_ mutant are at least as high as in *rec8Δ* mutants, which have a nearly intact checkpoint response (Fig 4D). Moreover, DSB formation was not further reduced in a *red1*_*ycs4S*_ *rec8Δ* double mutant (Fig 5A). Accordingly, DSB levels of the double mutant remained considerably higher than in *red1Δ* mutants, as indicated by pulse-field gel analysis of chromosome VIII (S6C and S6D Fig).

It is possible that *dmc1Δ rad51Δ red1*_*ycs4S*_ mutants fail to efficiently activate the checkpoint arrest because of a failure to produce sufficient ssDNA. ssDNA coated with the ssDNA-binding protein RPA is the major signal that activates the checkpoint kinase ATR/Mec1 [67]. To investigate the amount of ssDNA formed by hyper-resection in checkpoint-activated *dmc1Δ rad51Δ* mutants, we used Southern blotting to analyze the *CCT6* hotspot, which exhibits approximately 70% of wild-type DSB levels in all three mutants (Fig 5C and 5D). Resection can be seen at this hotspot as a smearing of the DSB band that progressively shifts to faster migrating species, corresponding to resection less than 5kb from the DSB site (Fig 5C and 5E). Resection is also detectable as fragments that are larger than the parental band (Fig 5C, Res1-4). These larger fragments are caused by the inability of a restriction enzyme to digest ssDNA once resection tracts have passed the respective restriction sites [40].

At 3h after induction of meiosis, all three mutants exhibited more extensive smearing of the DSB band compared to *dmc1Δ rad51Δ* control (Fig 5E), indicating more extensive resection. Moreover, the signal of the DSB band diminished in the mutants at later time points, as slower migrating fragments became apparent. Resection product Res4 (Fig 5C), which is produced when resection results in an ssDNA tract of at least 8kb, became detectable in *red1*_*ycs4S*_ *dmc1Δ rad51Δ* and *rec8Δ dmc1Δ rad51Δ* mutants 5h after induction of meiosis and increased in intensity over time (Fig 5F), suggesting that resection tracts persist in the *red1*_*ycs4S*_ *dmc1Δ rad51Δ* mutants even though a large portion of these cells have progressed out of meiotic prophase (Fig 4D). The Res4 signal in the *red1*_*ycs4S*_ *rec8Δ dmc1Δ rad51Δ* mutant appeared earlier and at a higher levels than either of the single mutants (Fig 5F), suggesting that hyper-resection is faster and more sustained. These experiments are consistent with previous analyses showing that loss or *RED1* or *REC8* function leads to more extensive resection [40,41], and argue that insufficient resection is not the cause of the observed bypass of the checkpoint arrest by *red1*_*ycs4S*_.

### Extended checkpoint arrest requires higher Red1 levels than faithful chromosome assortment

**Fig 6:**
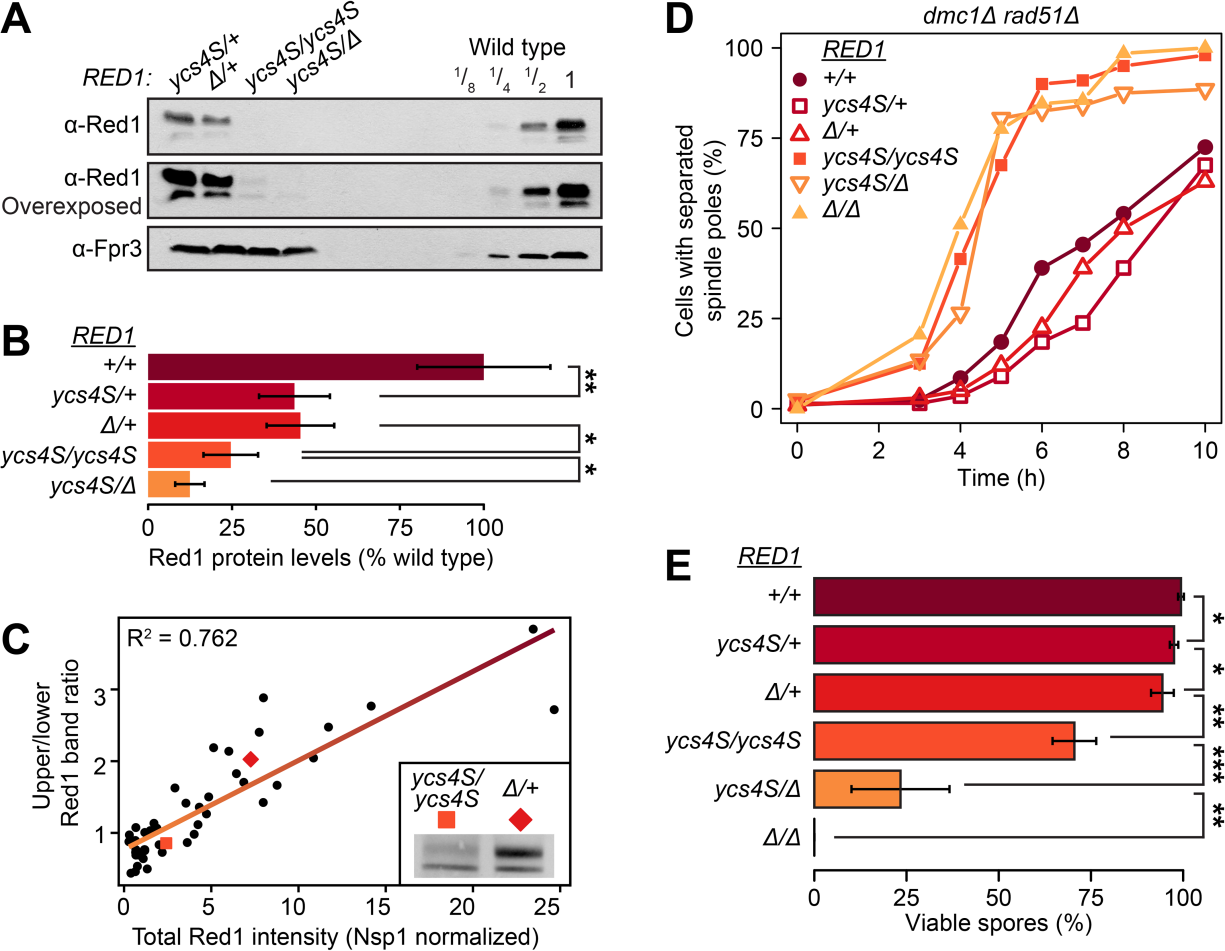
Checkpoint arrest and spore viability require different amounts of Red1 protein. **(A)** Semiquantitative western blot of a Red1 dosage series. Red1 levels of 2h samples from *red1*_*ycs4S*_*/RED1* (*ycs4S/+*, H8218), *red1Δ/RED1* (H8220, *Δ/+*), *red1*_*ycs4S*_*/red1*_*ycs4S*_ (*ycs4S/ycs4S*, H7011), and *red1*_*ycs4S*_ */red1Δ* (*ycs4S/Δ*, H8219) were compared to a 2-fold serial dilution of a whole-cell extract from wild type (*+/+*, H7797) from the same time course. Fpr3 was used as loading control. **(B)** Quantitative measurement of total Red1 protein levels in the dosage series relative to Nsp1 at 2h and 3h, n = 3 per time point. Error bars: S.E.M. *: p-value < 0.05, **: < 0.005, paired Student *t*-test. **(C)** Ratio of upper (modified) band to lower (unmodified) band correlates with Red1 protein levels. Red1 levels were normalized against Nsp1. Quantifications come from the gels used in the creation of Fig 1B and Fig 6B. Linear regression of the data is shown along with adjusted R^2^. Examples of two points are shown in the inset. **(D)** Spindle pole separation of *dmc1Δ rad51Δ* (closed circle, H7076), *dmc1Δ rad51Δ red1*_*ycs4S*_*/RED1* (open square, H8467), *dmc1Δ rad51Δ red1Δ/RED1* (open upward-facing triangle, H8494), *dmc1Δ rad51Δ red1*_*ycs4S*_*/red1*_*ycs4S*_ (closed squared, H7088), *dmc1Δ rad51Δ red1*_*ycs4S*_*/red1Δ* (open downward-facing triangle, H8504), and *dmc1Δ rad51Δ red1Δ/red1Δ* (closed upward-facing triangle, H6023), n = 200. **(E)** Spore viability of the Red1 dosage series and *red1Δ/red1Δ* (H8098), n>100. *: p-value < 0.05, **: < 0.005, ***: < e^-5^ Student *t*-test. Samples range in color from dark purple to pale orange as they transition from highest Red1 levels to lowest.

The phenotypes described above suggest that different meiotic processes are differentially affected by changes in Red1 dosage. Specifically, the Red1 levels associated with the *red1*_*ycs4S*_ mutant strain support sufficient DSB formation and homolog-directed crossover repair to yield nearly 70% viable spores (Fig 2B), yet are unable to trigger any detectable prophase delay in response to unrepaired DSBs (Fig 4D). To test this differential sensitivity in a more systematic manner, we took advantage of the *cis*-encoded reduction of Red1 protein levels of the *red1*_*ycs4S*_ strain to create a Red1 dosage series (Fig 6A). Quantitative western analysis indicated that *red1*_*ycs4S*_*/RED1* and *red1Δ/RED1* mutants have approximately 50% Red1 protein compared to wild type (Fig 6B), an estimate supported by direct comparison with a titration series of wild-type protein extracts (Fig 6A). The homozygous mutant *red1*_*ycs4S*_*/red1*_*ycs4S*_ strain and the *red1Δ/ red1*_*ycs4S*_ strain have approximately 25% and 15% of wild type Red1 protein, respectively. Consistent with the data shown in Fig 1A, increased Red1 protein levels correlated well with an increased proportion of the slower migrating phosphorylated form of Red1 (R^2^ = 0.762, Fig 6C), suggesting that Red1 phosphorylation is stimulated by Red1 abundance.

Analysis of this dosage series revealed that strains expressing at least 50% of wild type Red1 protein trigger a meiotic delay in a *dmc1Δ rad51Δ* background (Fig 6D). By contrast, no delay is observed in strains with equal or less than 25% Red1 protein. We note that a phosphorylation-deficient *red1* mutant fully maintains a *dmc1Δ* arrest [54], indicating that the observed loss of arrest activity is not caused by the dosage-dependent loss of Red1 phosphorylation. Importantly, despite the loss of checkpoint-dependent arrest activity, spore viability of *red1*_*ycs4S*_*/red1*_*ycs4S*_ mutants remains around 70%, with only mild meiosis I non-disjunction (Fig 6E, S7A–S7C Fig), suggesting that the ability of the meiotic checkpoint network to promote homolog-directed crossover formation remains at least partially active at around 25% Red1 levels. This interpretation is consistent with analysis of the *HIS4LEU2* locus, revealing a partial block to sister repair and only a limited reduction in crossover levels in this mutant [62]. Indeed, even 15% Red1 levels of wild type support substantial spore viability, unlike *red1Δ* or *hop1Δ* null mutants, which do not form viable spores [33,34] (Fig 6E), or homolog bias-defective *hop1-scd* mutants, which form less than 7% viable spores [45]. These data indicate that mounting a meiotic prophase delay requires substantially higher doses of Red1 than promoting largely faithful meiotic chromosome segregation.

In an attempt to investigate DSB formation and resection cytologically in this dosage series, we analyzed chromosome spreads of *dmc1Δ rad51Δ* mutants using an antibody against the RPA subunit Rfa2. Spread nuclei of all strains analyzed exhibited foci and short tracks of Rfa2 indicative of resected DSBs. However, cells expressing lower levels of Red1 had a higher probability of accumulating large clumps of Rfa2 in low-DAPI regions of the nucleus (S7D Fig). These Rfa2 aggregates do not co-localize with Zip1 aggregates and are also formed at high frequency in resection-defective *rad50S* mutants (S7E Fig), indicating that they are not representative of ssDNA exposed at Spo11-dependent DSB ends. RPA aggregates have been reported in other meiotic DSB repair mutants, including *rad52Δ* [68], but their significance remains unclear. We note, however, that their appearance correlated well with loss of spore viability in the *RED1* dosage series.

### Chromosome synapsis does not require abundant Red1 on lateral elements

**Fig 7:**
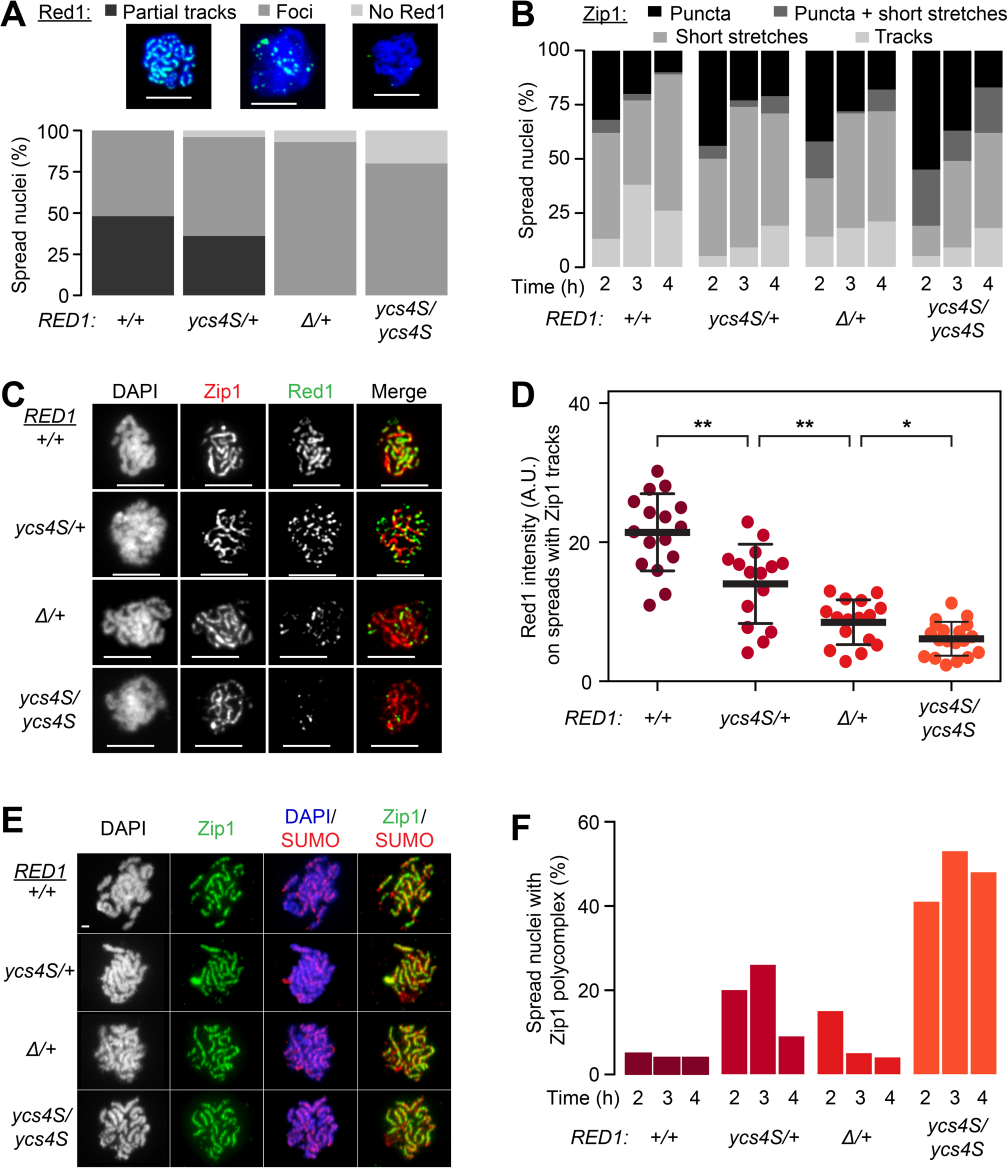
Chromosome synapsis occurs with minimal Red1 protein. **(A)** Quantification of Red1 binding on spread chromosomes 4h after meiotic initiation from wild-type (*+/+*, H7797), *red1*_*ycs4S*_*/RED1* (*ycs4S/+*, H8218), *red1Δ/RED1* (H8220, *Δ/+*), and *red1*_*ycs4S*_*/red1*_*ycs4S*_ (*ycs4S/ycs4S*, H7011) strains, n = 100. Only nuclei with detectable Zip1 staining were included. Red1 binding was classified into: “No Red1”, “Only Red1 foci”, and “Partial Red1 tracks”. Scale bars on spreads are 5μm. **(B)** Zip1 binding on chromosome spreads of wild-type, *red1*_*ycs4S*_*/RED1*, *red1Δ/RED1*, and *red1*_*ycs4S*_*/red1*_*ycs4S*_ strains, n = 100. Zip1 pattern was classified into: “Puncta”, “Puncta + short stretches”, “Short stretches”, and “Tracks”. **(C)** Example images of Red1 binding patterns on spreads with Zip1 tracks from **(B)**. Zip1 is colored red, while Red1 is colored green. Scale bars on spreads are 5μm. **(D)** Red1 intensity levels on chromosome spreads with Zip1 tracks, measured as described for Fig 3C, n>15. Error bars: S.D. *: p-value < 0.05, **: p-value < 0.005 Wilcox test. Samples range in color from dark purple to orange as they transition from highest Red1 level to lowest. **(E)** Representative images of SUMO binding patterns on spreads with Zip1 tracks from **(B)**. Zip1 is colored green, DAPI is colored blue, and SUMO is colored red. Scale bars are 1μm. **(F)** Frequency of Zip1 polycomplex formation in the spreads from **(B)**. Scale bars on spreads are 5μm.

Red1 is also an important regulator of chromosome synapsis, as *red1Δ* mutants form little to no SC, although occasional cells with more elaborate SC structures can be observed [24]. Therefore, we investigated the formation of lateral and central elements of the SC in the Red1 dosage series by immunofluorescence analysis. Red1 binding patterns on chromosomes reflect the reduced Red1 levels, with only the wild-type and *red1*_*ycs4S*_*/RED1* strains exhibiting at least partial Red1 tracks on chromosome spreads (Fig 7A). By contrast, Zip1 deposition along meiotic chromosomes is only mildly affected by decreased *RED1* dosage. Linear Zip1 tracks formed even in *red1*_*ycs4S*_*/red1*_*ycs4S*_ cells at levels similar to wild type (Fig 7B). Spreads with Zip1 tracks in these mutants had almost no cytologically detectable Red1 (Fig 7C and 7D), indicating that the majority of Red1 along lateral elements is dispensable for Zip1 deposition. Moreover, Zip1 tracks co-localize with SUMO in all strains (Fig 7E), suggesting the formation of mature central elements of the SC [69]. We did note an increase in late leptotene/early zygotene nuclei, characterized by the co-existence of abundant Zip1 puncta (leptotene configuration) and short Zip1 stretches (zygotene configuration), in *red1*_*ycs4S*_*/red1*_*ycs4S*_ mutants (Fig 7B). This class is rarely observed at higher Red1 dosage, and suggests that Zip1 deposition along chromosomes may be delayed at low Red1 levels. Consistent with this notion, we also observed an increasing incidence of Zip1 aggregates (polycomplexes; Fig 7F) as Red1 levels were reduced, although we note that polycomplex formation did not strictly correlate with Red1 levels, possibly because of the *Ycs4-12xMYC* tag in the *red1*_*ycs4S*_ background. These data suggest that abundant binding of Red1 to lateral elements is not required for the formation of cytologically normal SCs.

## Discussion

Here, we demonstrate that most of the phenotypes previously ascribed to the *ycs4S* allele are explained by a linked partial loss-of-function mutation in *RED1*. We took advantage of this allele to investigate the dependencies of meiotic prophase events on Red1 dosage. These experiments revealed substantial differences in the robustness of meiotic processes to reduced Red1 levels and indicate that at least some of the meiotic roles of Red1 are genetically separable.

### The genetic basis of *ycs4S* phenotypes

Our analyses show that the majority of *ycs4S* phenotypes can be explained by a linked point mutation in the *RED1* promoter that reduces *RED1* expression to ~25% of wild type. Deletion of *RED1* causes spore lethality because of numerous meiotic defects, including reduced DSB levels, hyper-resection, failure to block repair from the sister chromatid, defective chromosome synapsis, and an inability to arrest cells in response to persistent DSBs [33,38,40,41]. The *ycs4S* mutant recapitulates all of these phenotypes, albeit to varying extents (this study and [51,62]). Consistent with the *RED1* promoter mutation being the cause of most *ycs4S* phenotypes, a strain carrying this point mutation without the *12xMYC* tag on *YCS4* phenocopies the spore viability and arrest defects of the *ycs4S* mutant, while boosting *RED1* expression levels in *ycs4S* meiotic cells using a heterologous *HOP1* promoter essentially rescues the meiotic defects exhibited by *ycs4S* mutants.

The *YCS4-12xMYC* construct itself likely causes only minor meiotic effects. We observed a slightly higher rate of polycomplex formation in the *red1*_*ycs4S*_*/RED1* mutant compared to the *red1Δ/RED1* mutant, even though the *red1*_*ycs4S*_*/RED1* mutant appeared to have more Red1 protein on its chromosomes. This elevation in polycomplex formation may be attributable to the *YCS4-12xMYC* construct. In addition, Red1 protein expression is slightly reduced in the *red1*_*ycs4S*_ background even when *RED1* is placed under control of the *HOP1* promoter. Tagging *YCS4 de novo* with a C-terminal *MYC* tag does not affect spore viability (Fig 2B), suggesting that this effect may be caused by one of the additional SNPs in close linkage with the *red1* promoter mutation.

### Separation of Red1 roles in meiotic prophase

Our analyses indicate that reduced *RED1* levels impact some meiotic processes more severely than others. The ability to mount an arrest response to unrepaired breaks is abolished at a Red1 dosage of approximately 25% of wild-type levels. By contrast, DSB formation and SC assembly are only mildly affected, and sufficient inter-homolog crossovers form to support ~70% spore viability. The mild effect on axis-associated processes, such as DSB formation and SC assembly, indicates that the structural chromosome axis remains largely intact at these levels of Red1. This interpretation is consistent with the observation that *red1*_*ycs4S*_ mutants exhibit wild-type levels of chromosomal compaction [51], and that establishment of the crossover interference pattern, which requires SUMOylated Red1, is unaffected in *red1*_*ycs4S*_ mutants [70].

### The roles of Red1 in chromosome axis formation and checkpoint maintenance

The differential sensitivity of meiotic processes to altered Red1 levels may reflect differences in how long the presence of Red1 is needed to execute a given process. In particular, mounting a checkpoint delay or arrest that lasts for several hours is expected to require substantially longer residence time of Red1 on chromosomes than the formation of DSBs, which would require Red1 only until a DSB has formed. It is more difficult to explain the ability of cells with reduced Red1 levels to form synapsis-competent chromosome axes in this model, as stable axes are likely needed to support chromosome synapsis. It is possible, however, that Red1 is primarily involved in the formation chromosome axes and less in their structural maintenance. This model is consistent with the formation of axial structures in the absence of Red1 [33] and may explain the coexistence of leptotene and zygotene configurations in the same nucleus when Red1 levels are reduced (Fig 7B).

It is also possible that the differential sensitivity to altered Red1 levels reflects functionally distinct populations of Red1 on meiotic chromosomes that act in the assembly of DSB- and synapsis-competent chromosome axes and in checkpoint arrest maintenance, respectively. Indeed, Red1 not only interacts with multiple chromosomal proteins, including Rec8 and Hop1, but also multimerizes and is modified by both phosphorylation and SUMOylation, which could create functionally distinct forms of Red1 [32,37,42,54].

Finally, it is also possible that checkpoint signaling requires increased amounts of Red1 because of the need for signal amplification. This model is appealing because meiotic DSB resection tracts are kept relatively short by meiotic axis proteins [40,41], such that even resection tracts of irreparable breaks are maintained at lengths of about 1kb for several hours. By contrast, irreparable breaks in mitotic yeast cells are continuously resected, which is necessary for maintaining checkpoint activity of the CHK2-like kinase Rad53 [71,72]. In meiotic cells, Rad53 is largely inactive, as Spo11-dependent DSBs do not lead to Rad53 phosphorylation [73]. Instead, checkpoint maintenance is linked to the continued activity of CHK2-like kinase Mek1, which requires Red1 and Hop1 for activation [45,46,53,74]. Thus, the abundant binding of Red1 along chromosomes may amplify the binding and recruitment sites for Hop1 and Mek1 activation to support a sustained checkpoint signal without the associated risks of extensive resection. We note that Red1 is likely the limiting factor in this context because deletion of one copy of *HOP1* in a *red1*_*ycs4S*_*/RED1* strain background has no effect on spore viability (S7F Fig).

The notion that axial element-associated Red1 provides a means for signal amplification may also explain why *rec8Δ* mutants are able to mount a strong checkpoint arrest despite having little Red1 bound along chromosomes and showing no axial elements by electron microscopy [23]. Although DSB formation in *rec8Δ* mutants is largely restricted to genomic regions that are able to recruit Red1 independently of Rec8-cohesin [32], these mutants display large clumps of Red1 and Hop1 on chromosomes (Figs 3E, S3D). These clumps may support the checkpoint-dependent prophase arrest of these mutants despite the absence of detectable axial elements. Consistent with this interpretation, *rec8Δ red1*_*ycs4S*_ mutants do not form Hop1 clumps on chromosomes and fail to arrest.

## Methods

### Yeast strains and growth conditions

All strains had an SK1 background unless indicated otherwise. A complete list is located in S1 Table. The original *ycs4S* strain was a gift from D. Koshland [51], *SPO11-6HIS-3FLAG-loxP-KanMX-loxP* was provided by K. Ohta [48], and YPH499 was a gift from A. Strunnikov [75]. Gene disruption and tagging were carried out using a PCR-based protocol [76]. For *pHOP1-RED1* and *pHOP1-RED1*_*ycs4S*_, the *HOP1* promoter (-200 to -2) as defined by Vershon *et al*. [77] was placed in *pFA6a-kanMX6-pCLB2*, replacing *pCLB2*. It was inserted into the indicated genomes using Longtine F4 and RHop1 [AAT TCC TGA CCT TTC TGA AA] primers as described [76] replacing the 25bp immediately upstream of the *RED1* start codon with the *HOP1* promoter construct. To transfer the *red1-pG162A* mutation from the *ycs4S* background into a clean SK1 background, an *HphMX4* cassette [78] was inserted at -400 relative to the *RED1* start site. The entire promoter region (-600 to +90) was transferred into a wild-type SK1 background by PCR amplification and selection on hygromycin. Transfer of the mutation was confirmed by sequencing. An *HphMX4* insertion without associated promoter mutation was constructed in SK1 to serve as a wild-type control. Synchronous meiosis was induced as previously described [32].

### Western blotting

Whole cell extraction by trichloroacetic acid precipitation, SDS-polyacrylamide gel electrophoresis and western blotting were completed as described [79]. Hop1 and Red1 (Lot#16441) were detected using rabbit serum at 1:10,000 (kind gifts of N. Hollingsworth). Hop1 phosphorylation was detected using affinity-purified pT318-Hop1 antibody at 1:100 as described [80]. Phosphorylation of histone H3 threonine 11 (H3T11) was detected with MC83 rabbit antibody (Millipore) at 1:2000. Nsp1 was detected using 32D6 mouse antibody (ThermoFisher Scientific) at 1:2500. Pgk1 was detected using 22C5D8 mouse antibody (ThermoFisher Scientific) at 1:500. Fpr3 was detected with anti-rabbit serum (kind gift from J. Thorner) at 1:5000. For all westerns, the secondary antibody was either anti-mouse HRP or anti-rabbit HRP used at 1:5000 (GE Healthcare).

For quantitative westerns, Nsp1 was detected using 32D6 at 1:25,000 and Red1 was detected using an anti-Red1 antibody (Lot#16440; kind gift of N. Hollingsworth) at 1:4000. The secondary antibodies anti-rabbit IRDye800CW and anti-mouse IRDye680RD (Li-Cor Biosciences) were used at 1:10,000. Membranes were scanned on a Li-Cor Odyssey imager under non-saturating conditions. Data were quantified using pixel intensities with the Odyssey software according to the protocols of the manufacturer (Li-Cor Biosciences).

### Chromosome spreads

Meiotic nuclear spreads were performed as described [80]. Hop1 was detected using anti-Hop1 rabbit serum at 1:200 in blocking buffer and Alexa Fluor anti-rabbit (Jackson ImmunoResearch) at 1:200. Zip1 was detected using Zip1 yC-19 goat antibody (Santa Cruz Biotechnology) at 1:200 and anti-goat Cy3 at 1:200 (Jackson ImmunoResearch). Red1 was detected using anti-Red1 rabbit serum (Lot#16441) at 1:100 and Alexa Fluor anti-rabbit at 1:100. Rfa2 was detected using anti-Rfa2 rabbit serum (kindly provided by S. Brill) at 1:1000 and Alexa Fluor anti-rabbit at 1:200. Images were obtained as described [80] and analyzed using softWoRx 5.0 software. Scatterplots were created using Prism 6. For Fig 7E, affinity purified rabbit anti-Zip1 (raised at YenZym Antibodies, LLC, against a C terminal fragment of Zip1 as described [81]) was used at 1:100. Affinity purified guinea pig anti-SUMO was used at 1:200 (gift from G.S. Roeder [82]). Secondary antibodies Alexa Fluor 488 and Alexa Fluor 594 were used at 1:200 (Jackson ImmunoResearch). Microscopy and image processing were carried out using a Deltavision RT or a Deltavision Elite imaging system (Applied Precision) adapted to an Olympus IX17 microscope.

### Chromatin immunoprecipitation and Illumina sequencing

All cultures were collected at the 3-hr time point. Chromatin immunoprecipitation was performed as described [83]. Samples were immunoprecipitated with 2 μL of either anti-Red1 (Lot#16440) or anti-Hop1 serum per IP. For qPCR, input samples were diluted 50X more than ChIP samples. qPCR was completed as described previously [84]. Library preparation was completed as described [32]. Library quality was confirmed by Qubit HS assay kit and Agilent 2100 Bioanalyzer or 2200 TapeStation. 51-bp single-end sequencing was accomplished on an Illumina HiSeq 2500 instrument.

### Processing Illumina data

Sequencing reads were mapped to SacCer3 (S288C) using Bowtie [85]. The one condition adjusted was to only collect information about reads that mapped to a single position in the genome. Reads were also mapped to the SK1 genome (only allowing perfect matches) with similar results. Reads were extended towards 3’ ends to a final length of 150 bp using MACS-2.1.0 (https://github.com/taoliu/MACS) [86]. Normalization of read tag density was completed as described [32]. Plots shown are an average of two replicates. Datasets are available at GEO, accession number GSE87060.

### DSB and resection experiments

Genomic DNA for DSB hotspot analysis was purified as described [79]. For the *CCT6* hotspot, samples were digested with *Nsi*I and *Xho*I, and run on a 0.6% agarose gel for ~18 hr. The probe was created as described [8]. Pulse-field gel electrophoresis and Southern blotting were performed as described [3]. Hybridization signal was detected using a Typhoon FLA 9000. DSB levels on PFGE were estimated assuming a Poisson distribution as described [66]. Specifically, DSB levels were calculated as -ln(uncut_timepoint_/uncut_0h_).

### End-labeling of Spo11-oligonucleotide complexes

End-labeling of Spo11-oligonucleotide complexes was completed as described [65] with a few changes. In brief, 100mL SPO cultures were lysed with glass beads in 10% trichloroacetic acid. Lysed cells were centrifuged, and then resuspended in 1.5mL of 2% SDS, 0.5M Tris-HCl pH 8.0, 10mM EDTA, and 2% β-mercaptoethanol. After boiling the samples, soluble protein was diluted 2X in 2% Triton X100, 30mM Tris-HCl pH 8.0, 300mM NaCl, 2mM EDTA, and 0.02% SDS. Immunoprecipitation of the Spo11-oligo complexes was completed using 2.5μg of monoclonal mouse anti-Flag M2 antibody (Sigma). Precipitated complexes were end-labeled with 5μCi of [α-^32^P]dCTP and 5 units of terminal deoxynucleotidyl transferase (Enzymatics). End-labeled complexes were run on a Bolt 4–10% bis tris plus acrylamide gel (ThermoFisher Scientific), blotted onto a PVDF membrane using an iBlot2 gel transfer device (ThermoFisher Scientific), and visualized using a Typhoon FLA 9000 (GE Healthcare). Blots were also probed with mouse monoclonal anti-FLAG M2-HRP (Sigma).

### Other techniques

Spindle formation was followed by anti-tubulin immunofluorescence as described previously [87]. For each time point, 200 cells were classified based on whether or not they had undergone spindle pole separation, indicative of exit from meiotic prophase. Spore viability was determined by dissection of tetrads into individual spores unless otherwise stated. RNA was extracted as described [88]. Reverse transcription and qPCR was performed as previously described, but using RiboLock RNase inhibitor (Thermo Scientific) to prevent RNA degradation [84]. Primers used in this study are listed in S2 Table.

## Supporting information

S1 Fig **(A)** Fluorescence-based quantitative measurement of total Red1 protein levels in the *red1-pG162A* mutant strain (H9048) and its matched control (H9049) relative to Nsp1 at 2h, n = 3. Error bars: S.E.M. **(B)** Spore viability of hybrid diploid strains composed of YPH499 (H2389) and either SK1 *red1-pG162A* (H8919) or its matched SK1 wild-type control carrying only a marker insertion at position +400 upstream of the *RED1* ORF (H8901), n>60. Error bars: S.D. *: p-value: < 0.05, Student *t*-test.
(PDF)

S2 Fig **(A)** Hop1 chromosomal localization determined by ChIP-seq at 3h in wild-type (black, H119 & H6408), *red1*_*ycs4S*_ (cyan, H7011), *rec8Δ* (pink, H7660 & H7772), and *red1*_*ycs4S*_ *rec8Δ* (orange, H7661) strains on chromosomes III and VIII, n = 2. Large black circles indicate the positions of the centromeres. **(B)** Red1 and Hop1 distribution in the *red1*_*ycs4S*_ *rec8Δ* mutant compared to a mock (IgG only) control. Triangles indicate positions analyzed by ChIP-qPCR in S3B Fig. **(C)** Scatter plots of genome-wide ChIP-seq data (5kb averages) directly comparing two experiments from *red1*_*ycs4S*_ *rec8Δ* mutants. Left panel: biological replicates of Hop1 binding; middle panel: average Red1 and Hop1 binding as seen in **(B)**; right panel: Hop1 binding and the mock control.
(PDF)

S3 Fig **(A)** Red1 enrichment over input at 3h determined by ChIP-seq in wild-type (black, H119), *red1*_*ycs4S*_ (H7011), *rec8Δ* (H7660 & H7772), and *red1*_*ycs4S*_ *rec8Δ* (H7661) strains at six positions across the genome, n = 2. Peaks were defined as regions of Red1 and Hop1 binding in all four strains as identified by ChIP-seq. **(B)** qPCR analysis of Red1 binding at five peaks and one negative control shown in **(A)** in wild type (grey, H7797) and *red1*_*ycs4S*_ (cyan, H7011), *rec8Δ* (pink, H5187), and *red1*_*ycs4S*_ *rec8Δ* (orange, H7661) mutants, n = 3 biological replicates. Error bars: S.E.M. Peak 2, peak 5, and the negative control are marked in Fig 3D and S2B Fig using identifying triangles.
(TIF)

S4 Fig **(A)** Western analysis of Red1 and Hop1 protein levels of whole-cell extracts from wild type (H7797) and *red1*_*ycs4S*_ (H7011), *rec8Δ* (H5187), and *red1*_*ycs4S*_ *rec8Δ* (H7661) mutants induced to undergo synchronous meiosis. Phosphorylated Hop1 indicated by bracket. Pgk1 was used as loading control. **(B)** Fluorescence-based quantitative measurements of Red1 protein levels in *rec8Δ* and *red1*_*ycs4S*_ *rec8Δ* at 2h, n = 3. Total Red1 levels relative to loading control Nsp1. Error bars: S.E.M. **(C)** Representative images of the predominant cytological patterns of Red1 observed in *rec8Δ* and *red1*_*ycs4S*_ *rec8Δ* strains. Arrow indicates a Red1 clump. Scale bars are 5μm.
(PDF)

S5 Fig **(A)** Red1 protein levels of whole-cell extracts from *dmc1Δ rad51Δ* (H7076) and *dmc1Δ rad51Δ ycs4S* (H7088) as determined by western blotting at the indicated time points after meiotic induction. Fpr3 was used as loading control. **(B)** Fluorescence-based quantitative measurement of Red1 protein levels in *dmc1Δ rad51Δ* and *dmc1Δ rad51Δ ycs4S* relative to Nsp1 at 2h, 3h, and 4h, n = 3. Error bars: S.E.M. *: p-value < 0.05 paired Student *t*-test. **(C)** Spindle formation of *dmc1Δ red1-pG162A* (H9081, cyan open triangle) and its matched control (H9079, black open circle) strains to measure checkpoint activity, n = 200.
(PDF)

S6 Fig **(A)** DSB levels and patterns of chromosome XVI from gel in Fig 5B. Arrowhead indicates full-length chromosomes. **(B)** Quantifications of DSB levels on chromosome XVI, n = 2. Error bars: range. **(C)** Southern of pulse-field gel showing DSB patterns of chromosome VIII in *red1Δ*, *hop1Δ*, *rec8Δ*, and *red1*_*ycs4S*_ *rec8Δ* mutants relative to their controls in a *dmc1Δ rad51Δ* background. Arrowhead indicates full-length chromosomes. Strains (L-R) are H5594, H5995, H6023, H7076, H7161, and H6589. **(D)** DSB levels of gel in **(C)**, calculated from the level of remaining full-length chromosomes (see Methods).
(TIF)

S7 Fig **(A)** Sporulation efficiency of wild-type (H7797, *+/+*), *red1*_*ycs4S*_*/RED1* (*ycs4S/+*, H8218), *red1Δ/RED1* (H8220, *Δ/+*), *red1*_*ycs4S*_*/red1*_*ycs4S*_ (*ycs4S/ycs4S*, H7011), *red1*_*ycs4S*_ */red1Δ* (*ycs4S/Δ*, H8219), and *red1Δ/red1Δ* (*Δ/Δ*, H8098) strains, n = 200. **(B)** Percentage of tetrads from the strains in **(A)** that yielded 4, 3, 2, 1, or 0 viable colonies, n>100 tetrads per strain. **(C)** Viability of random spores from wild-type and *red1*_*ycs4S*_ strains. Equal numbers of spores from tetrads, dyads, and monads were assessed for viability, n = 80 spores per strain. **(D)** Quantification of Rfa2 clumps on spreads 4h after meiotic initiation in Red1 dosage series in *dmc1Δ rad51Δ* background, n = 100 (*RED1/RED1*: H7076, *red1*_*ycs4S*_*/RED1*: H8467, *red1Δ/RED1*: H8494, *red1*_*ycs4S*_*/red1*_*ycs4S*_: H7088, *red1*_*ycs4S*_*/ red1Δ*: H8504, and *red1Δ/red1Δ*: H6023). Only nuclei with detectable Zip1 staining were included. Arrow on example spread indicates: low-DAPI region, lack of Zip1 staining, and Rfa2 clump. Arrowhead points at Zip1 polycomplex. **(E)** Quantification of Rfa2 clumps on spread chromosomes from *rad50S* (H8099), and *rad50S red1*_*ycs4S*_*/red1*_*ycs4S*_ (H8096) strains, n = 200. **(F)** Spore viability in *red1*_*ycs4S*_*/RED1* and *red1*_*ycs4S*_*/RED1 hop1Δ/HOP1* mutants (H8866), n>100.
(PDF)

S1 Table Strain list. (DOCX)

S2 Table qPCR primer list. (DOCX)

S3 Table Data in figures. (XLSX)

S4 Table Data in supplemental figures. (XLSX)
