# Supplementary figures and images for: Reduced dosage of the chromosome axis factor Red1 selectively disrupts the meiotic recombination checkpoint in Saccharomyces cerevisiae
Source: PLoS Genet. 2017 Jul 26;13(7):e1006928. doi: 10.1371/journal.pgen.1006928 (PMC5549997; doi:10.1371/journal.pgen.1006928)

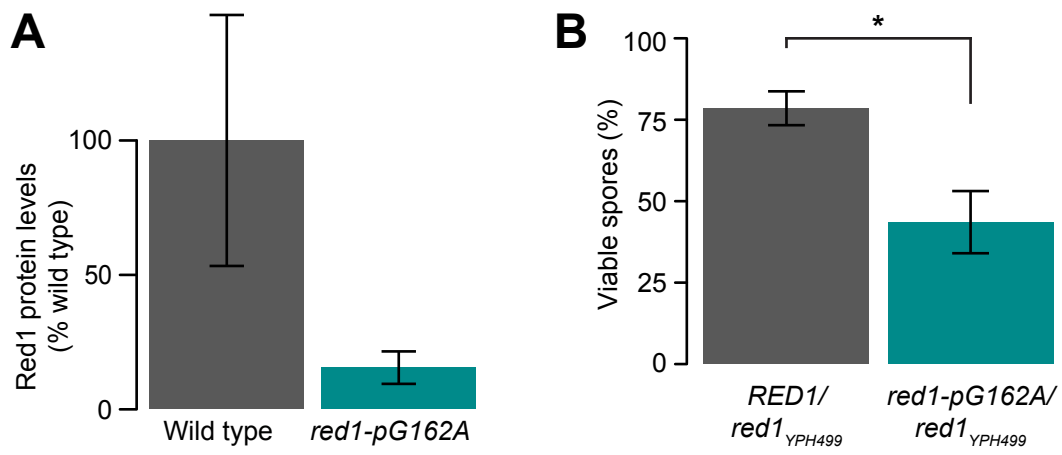

Supplement: S1 Fig — (A) Fluorescence-based quantitative measurement of total Red1 protein levels in the red1-pG162A mutant strain (H9048) and its matched control (H9049) relative to Nsp1 at 2h, n = 3. Error bars: S.E.M. (B) Spore viability of hybrid diploid strains composed of YPH499 (H2389) and either SK1 red1-pG162A (H8919) or its matched SK1 wild-type control carrying only a marker insertion at position +400 upstream of the RED1 ORF (H8901), n>60. Error bars: S.D. *: p-value: < 0.05, Student t-test. (PDF) [file pgen.1006928.s001.pdf]

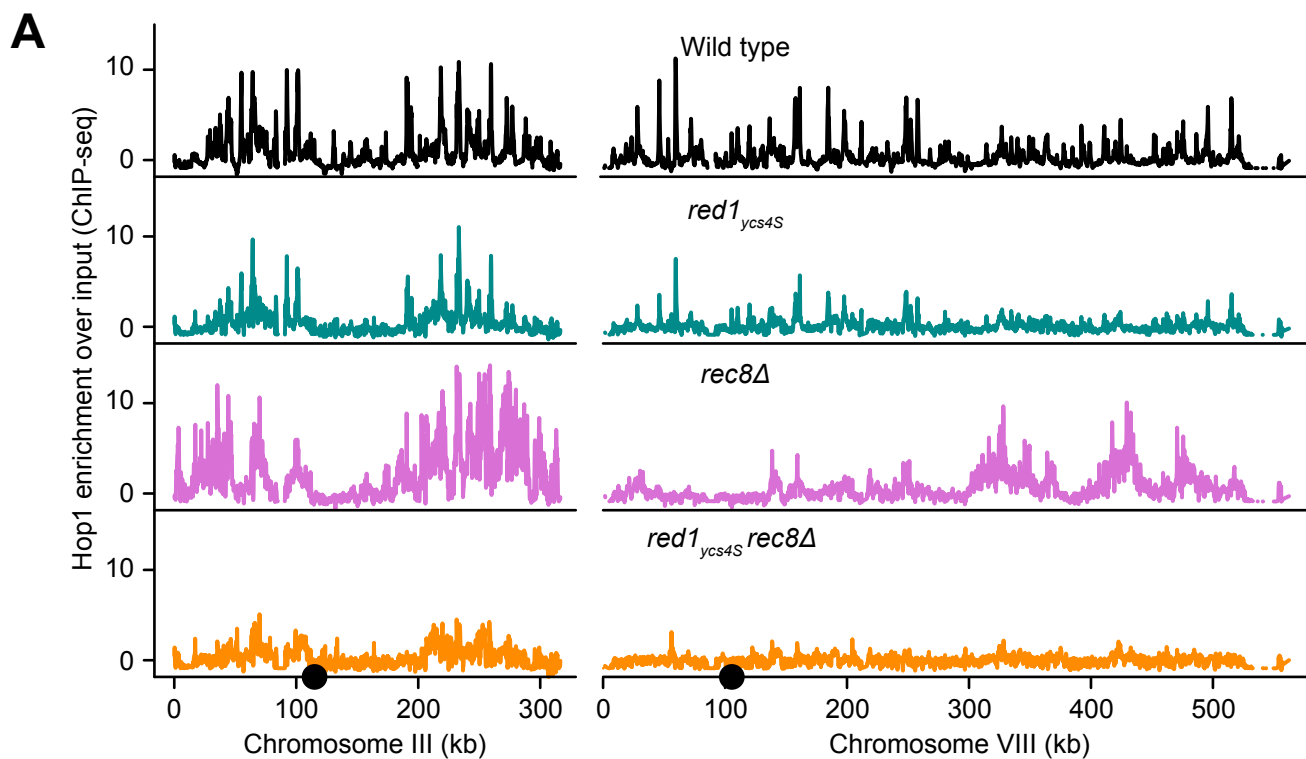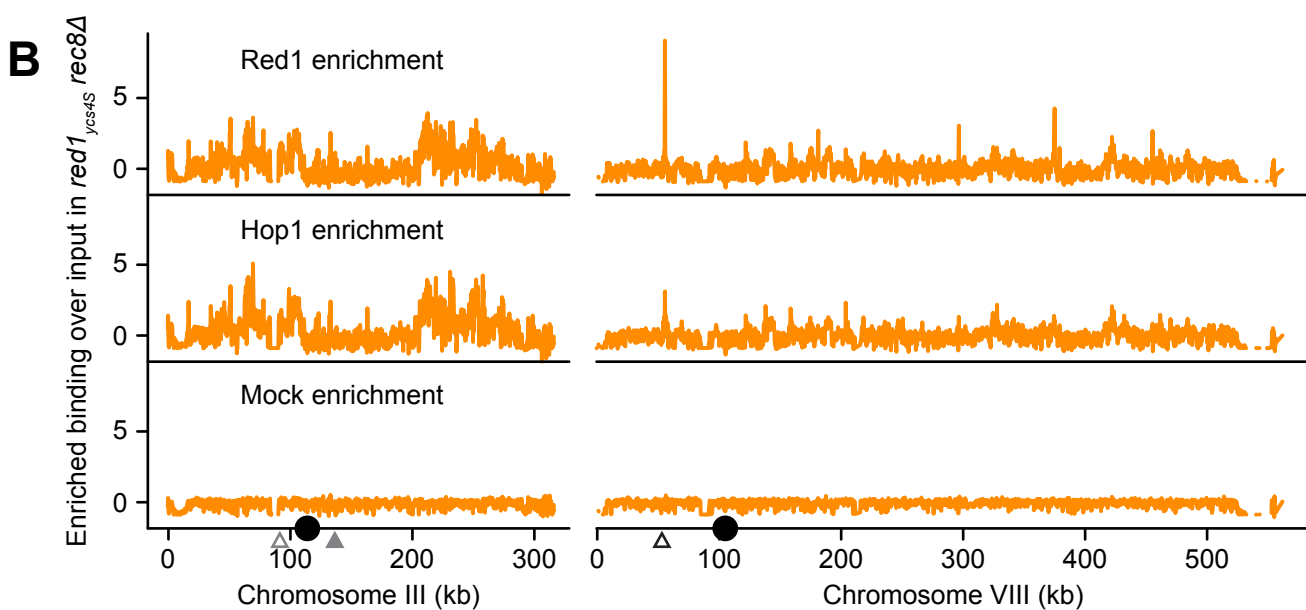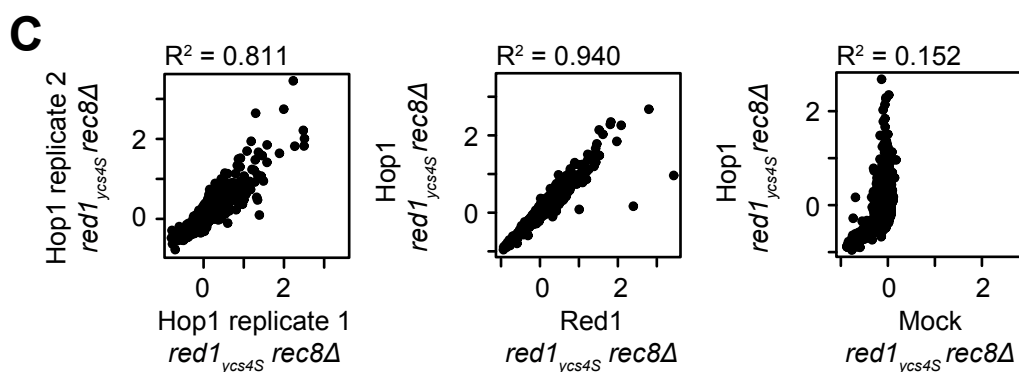

Supplement: S2 Fig — (A) Hop1 chromosomal localization determined by ChIP-seq at 3h in wild-type (black, H119 & H6408), red1ycs4S (cyan, H7011), rec8Δ (pink, H7660 & H7772), and red1ycs4S rec8Δ (orange, H7661) strains on chromosomes III and VIII, n = 2. Large black circles indicate the positions of the centromeres. (B) Red1 and Hop1 distribution in the red1ycs4S rec8Δ mutant compared to a mock (IgG only) control. Triangles indicate positions analyzed by ChIP-qPCR in S3B Fig. (C) Scatter plots of genome-wide ChIP-seq data (5kb averages) directly comparing two experiments from red1ycs4S rec8Δ mutants. Left panel: biological replicates of Hop1 binding; middle panel: average Red1 and Hop1 binding as seen in (B); right panel: Hop1 binding and the mock control. (PDF) [file pgen.1006928.s002.pdf]

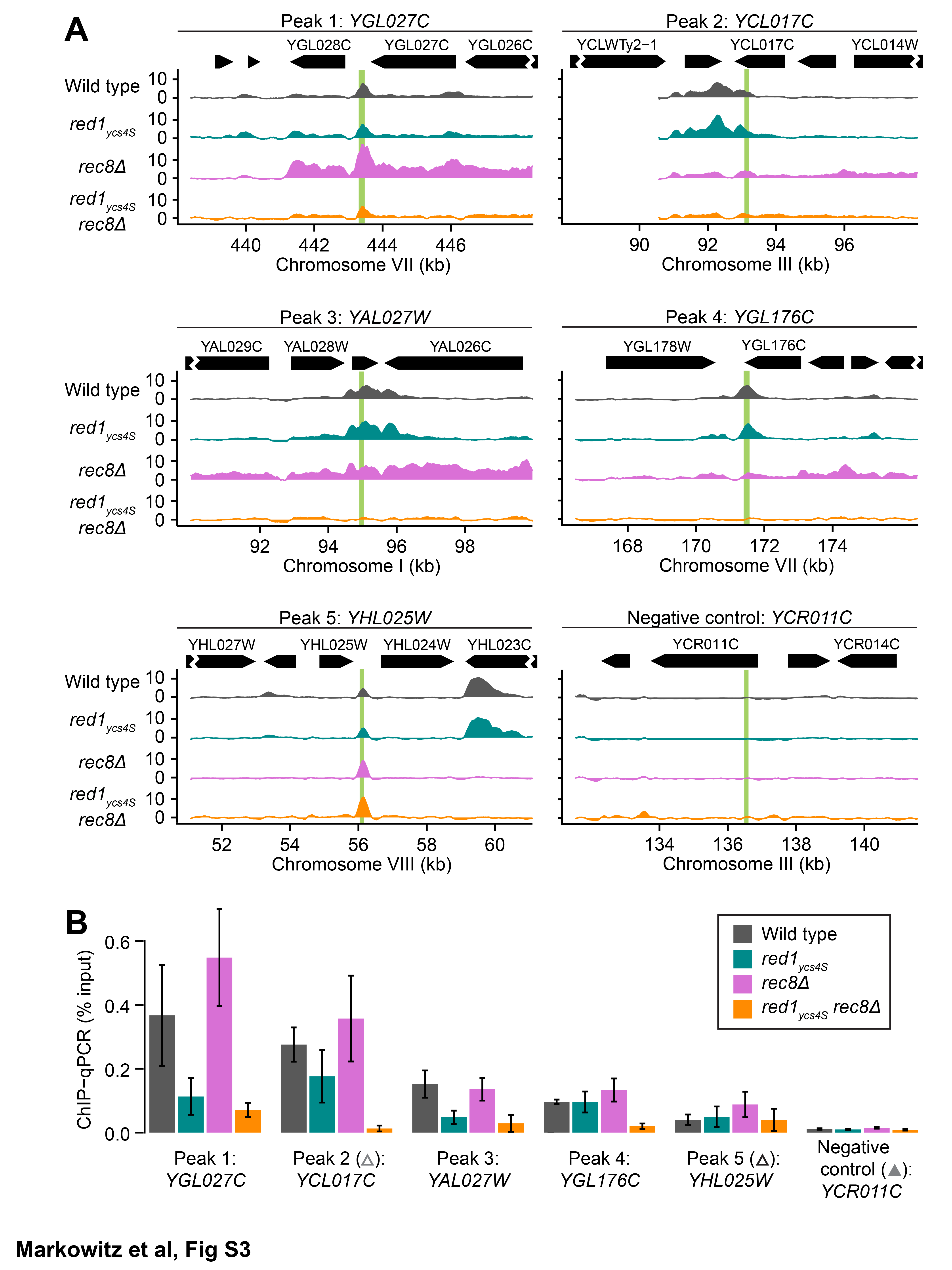

Supplement: S3 Fig — (A) Red1 enrichment over input at 3h determined by ChIP-seq in wild-type (black, H119), red1ycs4S (H7011), rec8Δ (H7660 & H7772), and red1ycs4S rec8Δ (H7661) strains at six positions across the genome, n = 2. Peaks were defined as regions of Red1 and Hop1 binding in all four strains as identified by ChIP-seq. (B) qPCR analysis of Red1 binding at five peaks and one negative control shown in (A) in wild type (grey, H7797) and red1ycs4S (cyan, H7011), rec8Δ (pink, H5187), and red1ycs4S rec8Δ (orange, H7661) mutants, n = 3 biological replicates. Error bars: S.E.M. Peak 2, peak 5, and the negative control are marked in Fig 3D and S2B Fig using identifying triangles. (TIF) [file pgen.1006928.s003.tif]

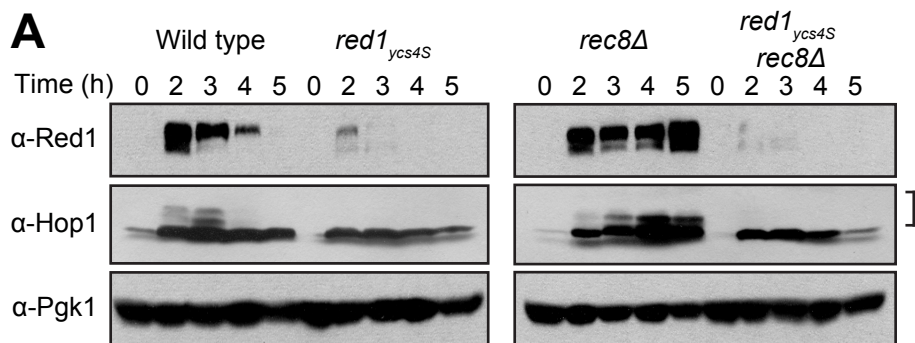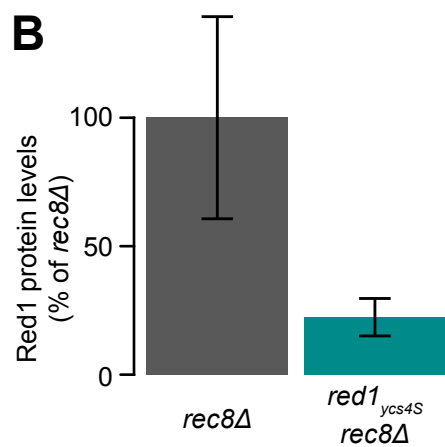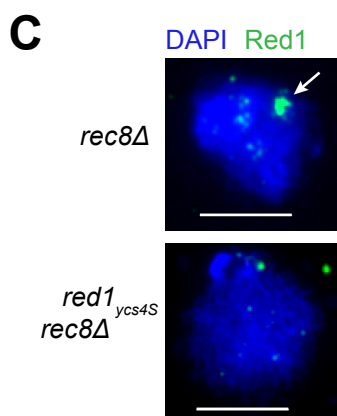

Supplement: S4 Fig — (A) Western analysis of Red1 and Hop1 protein levels of whole-cell extracts from wild type (H7797) and red1ycs4S (H7011), rec8Δ (H5187), and red1ycs4S rec8Δ (H7661) mutants induced to undergo synchronous meiosis. Phosphorylated Hop1 indicated by bracket. Pgk1 was used as loading control. (B) Fluorescence-based quantitative measurements of Red1 protein levels in rec8Δ and red1ycs4S rec8Δ at 2h, n = 3. Total Red1 levels relative to loading control Nsp1. Error bars: S.E.M. (C) Representative images of the predominant cytological patterns of Red1 observed in rec8Δ and red1ycs4S rec8Δ strains. Arrow indicates a Red1 clump. Scale bars are 5μm. (PDF) [file pgen.1006928.s004.pdf]

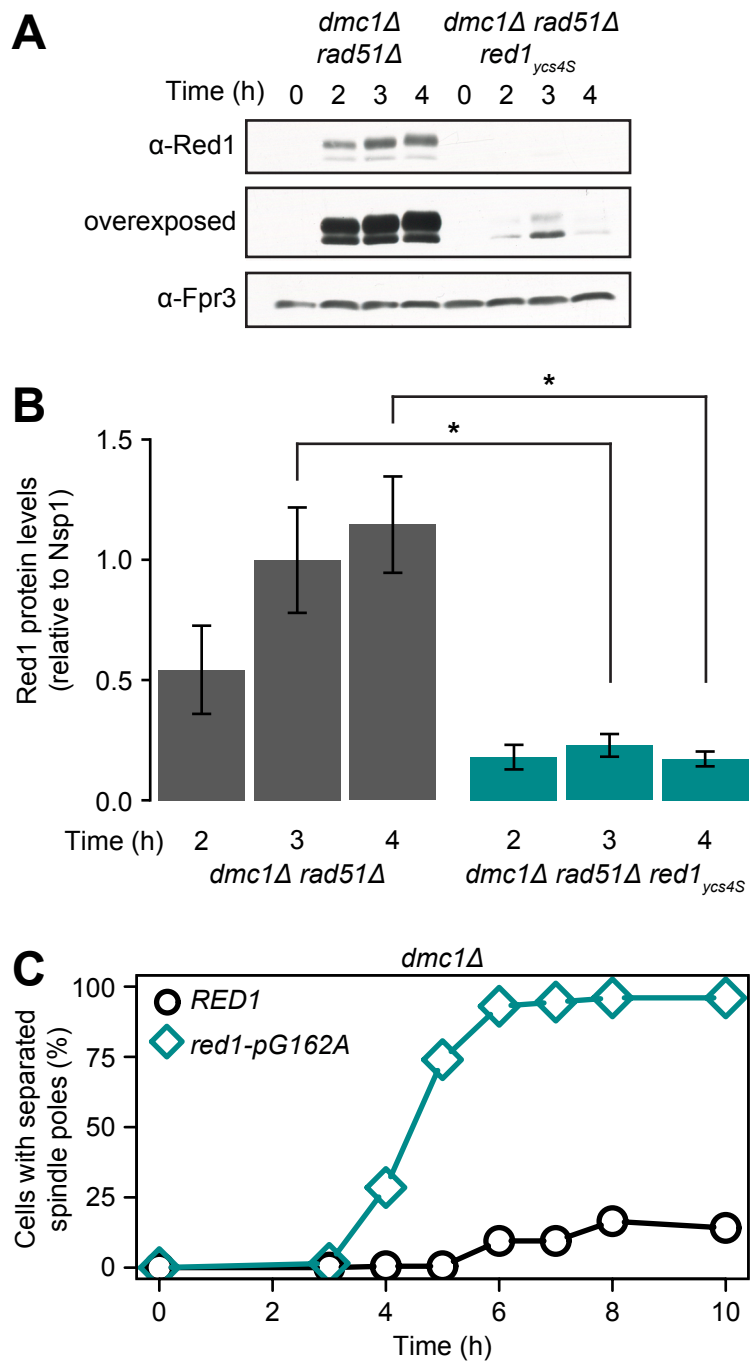

Supplement: S5 Fig — (A) Red1 protein levels of whole-cell extracts from dmc1Δ rad51Δ (H7076) and dmc1Δ rad51Δ ycs4S (H7088) as determined by western blotting at the indicated time points after meiotic induction. Fpr3 was used as loading control. (B) Fluorescence-based quantitative measurement of Red1 protein levels in dmc1Δ rad51Δ and dmc1Δ rad51Δ ycs4S relative to Nsp1 at 2h, 3h, and 4h, n = 3. Error bars: S.E.M. *: p-value < 0.05 paired Student t-test. (C) Spindle formation of dmc1Δ red1-pG162A (H9081, cyan open triangle) and its matched control (H9079, black open circle) strains to measure checkpoint activity, n = 200. (PDF) [file pgen.1006928.s005.pdf]

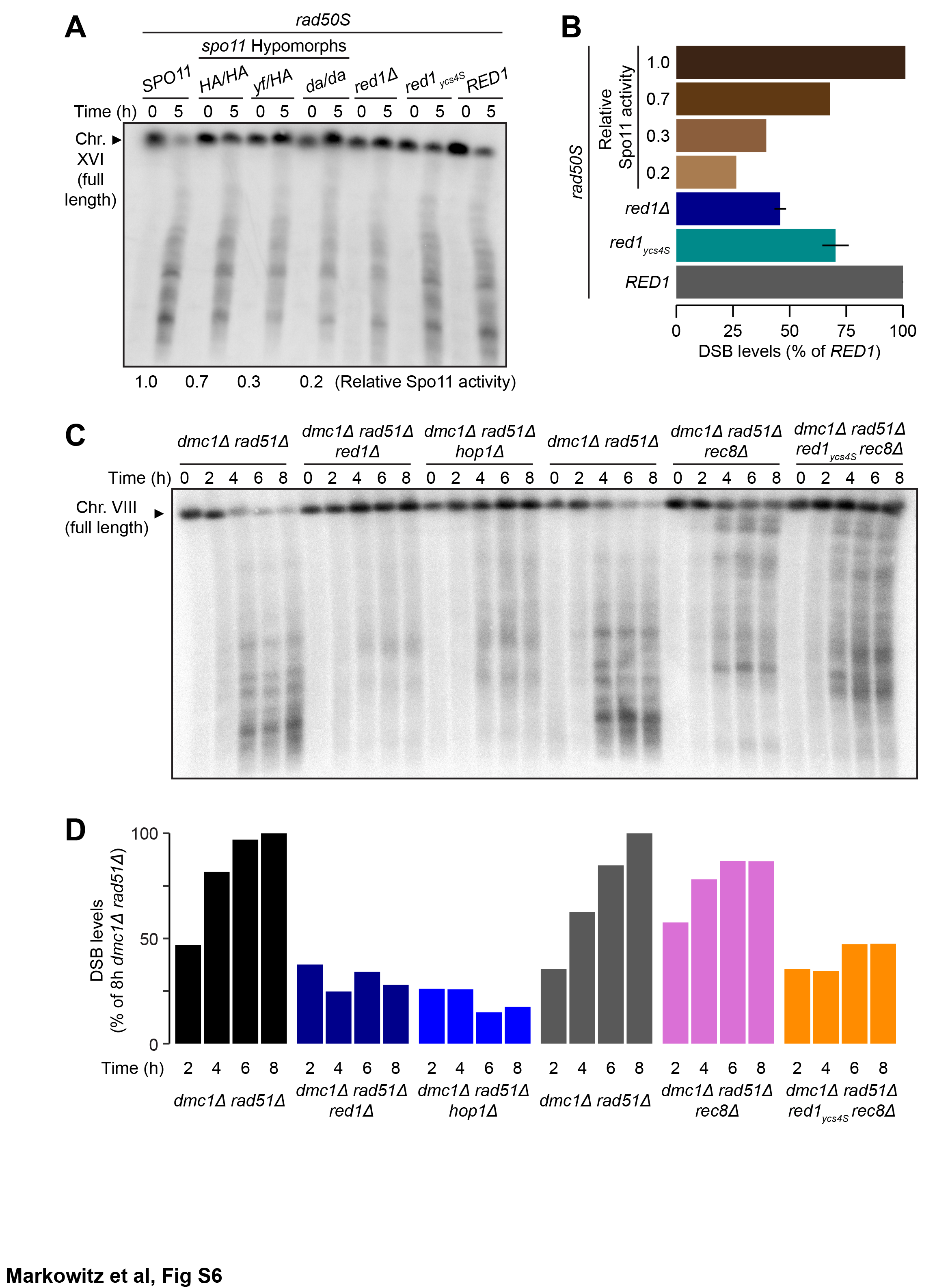

Supplement: S6 Fig — (A) DSB levels and patterns of chromosome XVI from gel in Fig 5B. Arrowhead indicates full-length chromosomes. (B) Quantifications of DSB levels on chromosome XVI, n = 2. Error bars: range. (C) Southern of pulse-field gel showing DSB patterns of chromosome VIII in red1Δ, hop1Δ, rec8Δ, and red1ycs4S rec8Δ mutants relative to their controls in a dmc1Δ rad51Δ background. Arrowhead indicates full-length chromosomes. Strains (L-R) are H5594, H5995, H6023, H7076, H7161, and H6589. (D) DSB levels of gel in (C), calculated from the level of remaining full-length chromosomes (see Methods). (TIF) [file pgen.1006928.s006.tif]

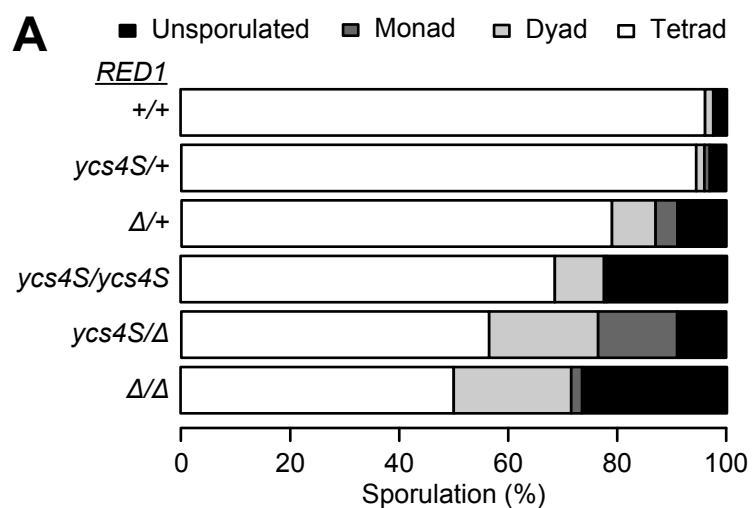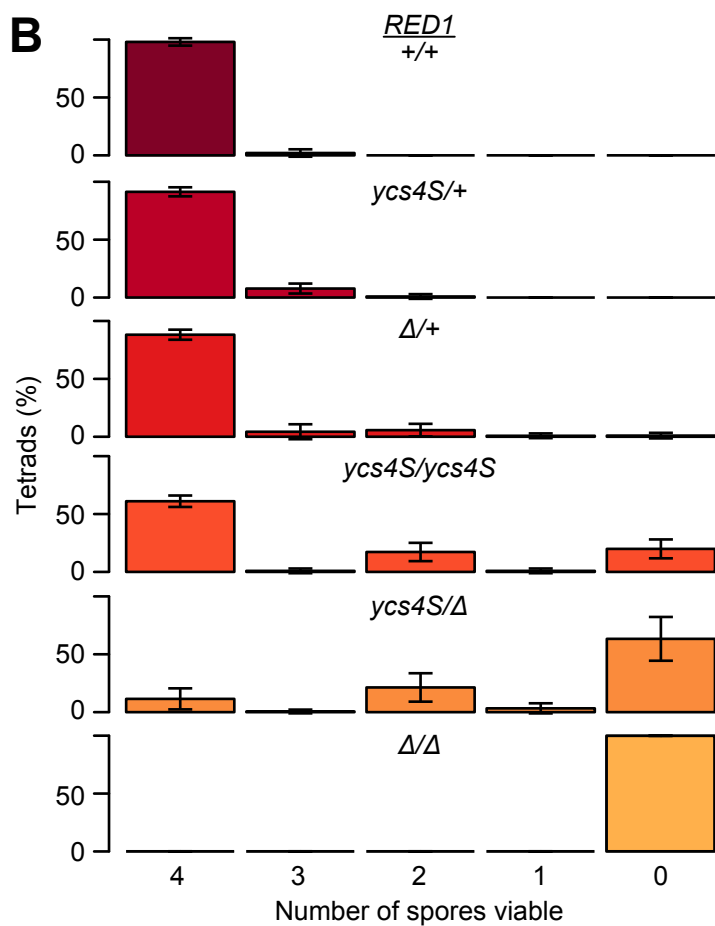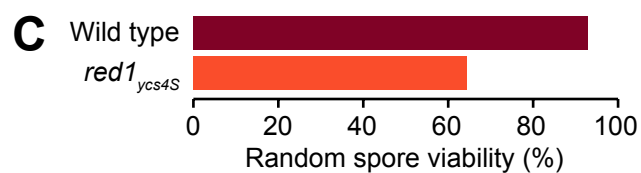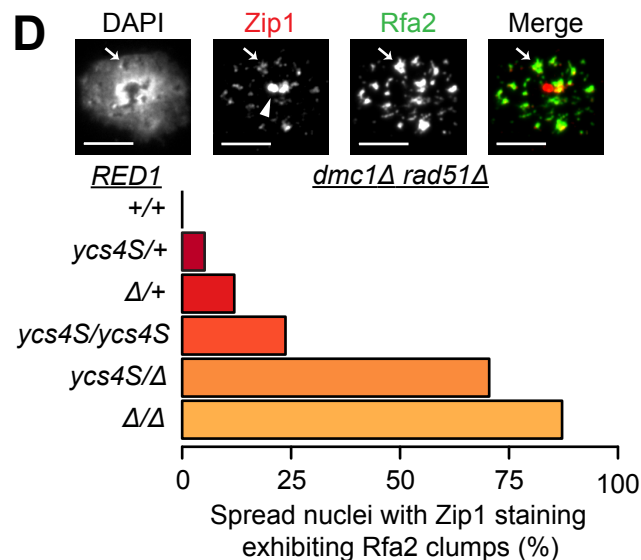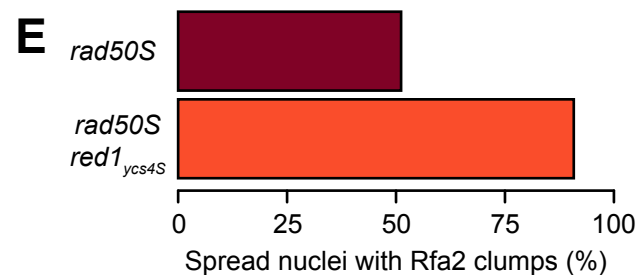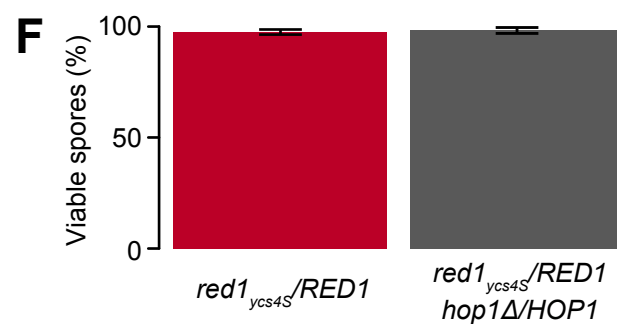

Supplement: S7 Fig — (A) Sporulation efficiency of wild-type (H7797, +/+), red1ycs4S/RED1 (ycs4S/+, H8218), red1Δ/RED1 (H8220, Δ/+), red1ycs4S/red1ycs4S (ycs4S/ycs4S, H7011), red1ycs4S /red1Δ (ycs4S/Δ, H8219), and red1Δ/red1Δ (Δ/Δ, H8098) strains, n = 200. (B) Percentage of tetrads from the strains in (A) that yielded 4, 3, 2, 1, or 0 viable colonies, n>100 tetrads per strain. (C) Viability of random spores from wild-type and red1ycs4S strains. Equal numbers of spores from tetrads, dyads, and monads were assessed for viability, n = 80 spores per strain. (D) Quantification of Rfa2 clumps on spreads 4h after meiotic initiation in Red1 dosage series in dmc1Δ rad51Δ background, n = 100 (RED1/RED1: H7076, red1ycs4S/RED1: H8467, red1Δ/RED1: H8494, red1ycs4S/red1ycs4S: H7088, red1ycs4S/ red1Δ: H8504, and red1Δ/red1Δ: H6023). Only nuclei with detectable Zip1 staining were included. Arrow on example spread indicates: low-DAPI region, lack of Zip1 staining, and Rfa2 clump. Arrowhead points at Zip1 polycomplex. (E) Quantification of Rfa2 clumps on spread chromosomes from rad50S (H8099), and rad50S red1ycs4S/red1ycs4S (H8096) strains, n = 200. (F) Spore viability in red1ycs4S/RED1 and red1ycs4S/RED1 hop1Δ/HOP1 mutants (H8866), n>100. (PDF) [file pgen.1006928.s007.pdf]
