# Supplementary material for: Reduced dosage of the chromosome axis factor Red1 selectively disrupts the meiotic recombination checkpoint in Saccharomyces cerevisiae
Source: PLoS Genet. 2017 Jul 26;13(7):e1006928. doi: 10.1371/journal.pgen.1006928 (PMC5549997; doi:10.1371/journal.pgen.1006928)
Supplement: S1 Table — (DOCX) [file pgen.1006928.s008.docx]

**Table S1. Strain list***

| Strain Name | Genotype | Figure |
| --- | --- | --- |
| **Wild-type background** | | |
| H7797 | *MATa/MATα, ho::LYS2/”, lys2/”, ura3/URA3, leu2::hisG/LEU2, his3::hisG/HIS3, trp1::hisG/TRP1* | 1,2,3,4,6,  7,S3,S4,S7 |
| H7011 | Same as H7797 except *red1_ycs4S_/red1_ycs4S_* | 1,2,3,4,6,  7,S2,S3,  S4,S7 |
| H8601 | Same as H7797 except *his4X::LEU2-URA3/”, ycs4-2::HIS3/ycs4-2::HIS3* | 1 |
| H8632 | Same as H7797 except *ycg1-2::Kan/ycg1-2::Kan* | 1 |
| H8849 | Same as H7797 except *KanMX6::pHOP1-RED1/KanMX6::pHOP1-RED1* | 2 |
| H8850 | Same as H7797 except  *KanMX6::pHOP1-RED1_ycs4S_/KanMX6::pHOP1-RED1_ycs4S_* | 2 |
| H9048 | Same as H7797 except *HphMX4::red1-pG162A/HphMX4::red1-pG162A* | 2,S1 |
| H9049 | Same as H7797 except *HphMX4::RED1/HphMX4::RED1* | 2,S1 |
| H9077 | Same as H7797 except  *YCS4-13xMYC::KanMX6/YCS4-13xMYC::KanMX6* | 2 |
| H5187 | Same as H7797 except *rec8Δ::HIS3MX6/rec8Δ::HIS3MX6* | 3,4,S3,S4 |
| H7661 | Same as H7797 except *red1_ycs4S_/red1_ycs4S_, rec8Δ::HIS3MX6/rec8Δ::HIS3MX6* | 3,4,S2,S3,S4 |
| H7660 | Same as H7797 except *SMC4-PK9::HIS3/SMC4-PK9::HIS3, rec8Δ::HIS3MX6/rec8Δ::HIS3MX6* | 3,S2,S3 |
| H119 | *MATa/MATα, ho::LYS2/”, lys2/”, ura3/”, leu2::hisG/”, his4B::LEU2/his4X::LEU2(Bam)-URA3, arg4-BglII/arg4-Nsp* | 3,S2,S3 |
| H7772 | Same as H119 except *rec8Δ::HIS3MX6/rec8Δ::HIS3MX6,*  *scc1Δ::SCC1-6HA::KanMX6/scc1Δ::SCC1-6HA::KanMX6* | 3,S2,S3 |
| H9082 | Same as H7797 except  *SPO11-6His-3FLAG-KanMX*/*SPO11-6His-3FLAG-KanMX* | 5 |
| H9083 | Same as H9082 except *rec8Δ::HIS3MX6/rec8Δ::HIS3MX6* | 5 |
| H9084 | Same as H9082 except *red1_ycs4S_/red1_ycs4S_* | 5 |
| H9085 | Same as H9082 except *red1_ycs4S_/red1_ycs4S_, rec8Δ::HIS3MX6/rec8Δ::HIS3MX6* | 5 |
| H8098 | Same as H7797 except *red1Δ::KanMX4/red1Δ::KanMX4* | 6,S7 |
| H8218 | Same as H7797 except *red1_ycs4S_*/*RED1* | 6,7,S7 |
| H8220 | Same as H7797 except *red1Δ::KanMX4/RED1* | 6,7,S7 |
| H8219 | Same as H7797 except *red1_ycs4S_*/*red1Δ::KanMX4* | 6,S7 |
| H6408 | Same as H7797 except *SMC4-PK9::HIS3/SMC4-PK9::HIS3* | S2 |
| H8866 | Same as H7797 except *red1_ycs4S_*/*RED1, hop1Δ::LEU2/HOP1* | S7 |
| ***dmc1Δ rad51Δ* background** | | |
| H7076 | Same as H119 except *dmc1Δ::ARG4/”, rad51Δ::HIS3/”* | 4,5,6,S5,  S6,S7 |
| H7088 | Same as H7076 except *red1_ycs4S_/red1_ycs4S_* | 4,5,6,  S5,S7 |
| H7161 | Same as H7076 except *rec8Δ::HIS3MX6/rec8Δ::HIS3MX6* | 4,5,S6 |
| H6589 | Same as H7076 except *red1_ycs4S_/red1_ycs4S_, rec8Δ::HIS3MX6/rec8Δ::HIS3MX6* | 4,5,S6 |
| H8851 | Same as H7076 except *KanMX6::pHOP1-RED1/KanMX6::pHOP1-RED1* | 4 |
| H8852 | Same as H7076 except  *KanMX6::pHOP1-RED1_ycs4S_/KanMX6::pHOP1-RED1_ycs4S_* | 4 |
| H9078 | *MATa/MATα, ho::LYS2/”, lys2/”, ura3/”, leu2::hisG/”, his3::hisG/”,*  *his4X::LEU2(Bam)-URA3/”, dmc1Δ::ARG4/”, rad51Δ::HIS3/”, HphMX4::RED1/HphMX4::RED1* | 4 |
| H9080 | *MATa/MATα, ho::LYS2/”, lys2/”, ura3/”, leu2::hisG/”, his3::hisG/”,*  *his4X::LEU2(Bam)-URA3/”, dmc1Δ::ARG4/”, rad51Δ::HIS3/”, HphMX4::red1-pG162A/HphMX4::red1-pG162A* | 4 |
| H8467 | Same as H7076 except *red1_ycs4S_*/*RED1* | 6,S7 |
| H8494 | Same as H7076 except *red1Δ::KanMX4/RED1* | 6,S7 |
| H8504 | Same as H7076 except *red1_ycs4S_*/*red1Δ::KanMX4* | 6,S7 |
| H5594 | *MATa/MATα, ho::LYS2/”, lys2/”, ura3/”, leu2::hisG/”, his4B::LEU2/”,*  *arg4-BglII/”, dmc1Δ::ARG4/”, rad51Δ::HIS3/”* | S6 |
| H5995 | Same as H5594 except  *hop1Δ::URA3/hop1Δ::URA3* | S6 |
| H6023 | Same as H5594 except *red1Δ::KanMX4/red1Δ::KanMX4* | 6,S6,S7 |
| ***dmc1Δ* background** | | |
| H9079 | *MATa/MATα, ho::LYS2/”, lys2/”, ura3/”, leu2::hisG/”, his3::hisG/”,*  *his4X::LEU2(Bam)-URA3/”, dmc1Δ::ARG4/”, HphMX4::RED1/HphMX4::RED1* | S5 |
| H9081 | *MATa/MATα, ho::LYS2/”, lys2/”, ura3/”, leu2::hisG/”, his3::hisG/”,*  *his4X::LEU2(Bam)-URA3/”, dmc1Δ::ARG4/”,*  *HphMX4::red1-pG162A/HphMX4::red1-pG162A* | S5 |
| ***rad50S* background** | | |
| H8099 | *MATa/MATα, ho::LYS2/”, lys2/”, ura3/”, leu2::hisG/”, his4B::LEU2/”,*  *arg4-BglII/”, rad50S::URA3/”* | 5,S6,S7 |
| H8096 | Same as H8099 except *red1_ycs4S_/red1_ycs4S_* | 5,S6,S7 |
| H8058 | Same as H8099 except *red1Δ::KanMX4/red1Δ::KanMX4* | 5,S6 |
| H9202 | *MATa/MATα, ho::LYS2/”, lys2/”, ura3/”, leu2/”, arg4-NspI/ARG4, nuc1Δ::LEU2/”, rad50S::URA3/”* [ SKY48 x SKY1662 ] | 5,S6 |
| H9203 | Same as 9202 except  *spo11-HA3-His6::kanMX4/spo11-HA3-His6::kanMX4*  [ SKY1663 x SKY1664 ] | 5,S6 |
| H9204 | Same as 9202 except  *spo11-HA3-His6::kanMX4/spo11(Y135F)-HA3-His6::kanMX4*  [ SKY1665 x SKY1664 ] | 5,S6 |
| H9205 | Same as 9202 except *spo11(D290A)-HA3-His6::kanMX4/ spo11(D290A)-HA3-His6::kanMX4* [ SKY9193 x SKY1668 ] | 5,S6 |
| **Haploids** | | |
| H2389 | *MATa, lys2, ura3, ade2, trp1, his3, leu2, bar1-Δ, pep4::HIS3, SMC2-12His:3HA::LEU2* [YPH499 strain background] | S1 |
| H8901 | *MATα, ho::LYS2, lys2, HphMX4::RED1* | S1 |
| H8918 | *MATα, ho::LYS2, lys2, HphMX4::red1-pG162A* | S1 |

* subscript *ycs4S* indicates that this marker is linked with *YCS4-12xMYC::HIS3* and the surrounding introgressed region from YPH499
