# Supplementary material for: Reduced dosage of the chromosome axis factor Red1 selectively disrupts the meiotic recombination checkpoint in Saccharomyces cerevisiae
Source: PLoS Genet. 2017 Jul 26;13(7):e1006928. doi: 10.1371/journal.pgen.1006928 (PMC5549997; doi:10.1371/journal.pgen.1006928)
Supplement: S2 Table — (DOCX) [file pgen.1006928.s009.docx]

**Table S2. Primers used for qPCR.**

| Locus | Forward/reverse | Sequence |
| --- | --- | --- |
| **Fig 1D** |  |  |
| *ACT1* | Forward | CAC TGC TGA AAG AGA AAT TGT C |
|  | Reverse | TTG ACC TTC ATG GAA GAT GGA |
| *RED1* | Forward | AGA CGA GAA AGC CCC TTT GG |
|  | Reverse | ATT CGA GCT GAA ACC CCC TC |
| *HOP1* | Forward | GCC GGA GTT GTT CAA AGA CG |
|  | Reverse | GCC GCC GAC AAA ACA GAA AG |
| *REC8* | Forward | AAA ATG AAG GGC CGG TAG CC |
|  | Reverse | CGC GAT GTC CTC TTT GCA TTC |
| *ZIP1* | Forward | TCT GCT GCC ATT CAC TCT CC |
|  | Reverse | TTG GAT TGG GGA AGG GTT GG |
| **S3B Fig** |  |  |
| Peak 1: *YGL027C* | Forward | CGA TGC GGC TGT GAG AAA AAG |
|  | Reverse | GAA AAG ACC TCA CTT CGC AAC |
| Peak 2: *YCL017C* | Forward | TCC ACG TAG GCG AAA GAA AC |
|  | Reverse | TTG ACA ACG ACC AAG CTC AC |
| Peak 3: *YAL027W* | Forward | GTG ACG AGG TCA AGG AGG AC |
|  | Reverse | AGC AAA AAG TTT GGG CAG AA |
| Peak 4: *YGL176C* | Forward | CAA AAA TCT CGG TGG GCT AA |
|  | Reverse | TGT TTG ATG CTG TGG AAC CT |
| Peak 5: *YHL025W* | Forward | ATT CGT TGG TTG ATG TGG CG |
|  | Reverse | TGA GGC GGT GTT TAC GAC TG |
| Negative control:  *YCR011C* | Forward | GGT GAT GAT TGC TCT CTG CC |
|  | Reverse | CGT CAC AAT TGA TCC CTC CC |
